# Controlling Electron Spin Decoherence in Nd-based Complexes via Symmetry Selection

**DOI:** 10.1016/j.isci.2020.100926

**Published:** 2020-02-20

**Authors:** Jing Li, Lei Yin, Shi-Jie Xiong, Xing-Long Wu, Fei Yu, Zhong-Wen Ouyang, Zheng-Cai Xia, Yi-Quan Zhang, Johan van Tol, You Song, Zhenxing Wang

**Affiliations:** 1 Wuhan National High Magnetic Field Center & School of Physics, Huazhong University of Science and Technology, Wuhan 430074, P. R. China; 2 State Key Laboratory of Coordination Chemistry, School of Chemistry and Chemical Engineering, Nanjing University, Nanjing 210023, P. R. China; 3 National High Magnetic Field Laboratory, Florida State University, Tallahassee, FL 32310, USA; 4 Jiangsu Key Laboratory for NSLSCS, School of Physical Science and Technology, Nanjing Normal University, Nanjing 210023, P. R. China; 5 National Laboratory of Solid State Microstructures and Department of Physics, Nanjing University, Nanjing 210093, P. R. China

**Keywords:** Materials Property, Molecules, Quantum Chemical Calculations

## Abstract

Long decoherence time is a key consideration for molecular magnets in the application of the quantum computation. Although previous studies have shown that the local symmetry of spin carriers plays a crucial part in the spin-lattice relaxation process, its role in the spin decoherence is still unclear. Herein, two nine-coordinated capped square antiprism neodymium moieties [Nd(CO_3_)_4_H_2_O]^5–^ with slightly different local symmetries, *C*_1_ versus *C*_4_ (**1** and **2**), are reported, which feature in the easy-plane magnetic anisotropy as shown by the high-frequency electron paramagnetic resonance (HF-EPR) studies. Detailed analysis of the relaxation time suggests that the phonon bottleneck effect is essential to the magnetic relaxation in the crystalline samples of **1** and **2**. The 240 GHz Pulsed EPR studies show that the higher symmetry results in longer decoherence times, which is supported by the first principle calculations.

## Highlights

• Neodymium-based complexes show the spin decoherence without the magnetic dilution
• The higher structural symmetry results in longer spin decoherence times
• The phonon bottleneck effect induces the slow magnetization relaxation behaviors

## Graphical Abstract

**Figure:**
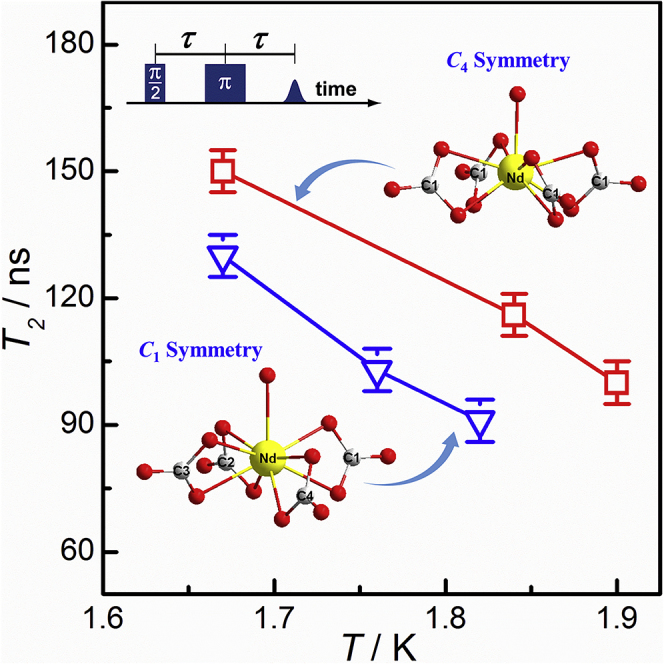

## Introduction

Single-molecule magnets (SMMs) (Sessoli et al., 1993) are promising candidates as the quantum bits (qubits), the basic building blocks of a quantum computer according to Leuenberger and Loss's proposal (Leuenberger and Loss, 2001), in which they show slow spin relaxation behaviors between the bistable ground states with an energy barrier. However, the large zero-field splittings of SMMs result in low population in high-energy levels at low temperatures, which hinders the application of SMMs as qubits (Takahashi et al., 2008, Takahashi et al., 2009, Takahashi et al., 2011, Wang et al., 2011). In recent years, there has been a drive to achieve smaller energy splittings using the mesoscopic spin states produced by hyperfine interactions between the electron and nuclear spins as a substitution, which are termed as qudits (Aguilà et al., 2014, Atzori et al., 2016a, Atzori et al., 2016b, Atzori et al., 2017, Fataftah et al., 2016, Graham et al., 2014, Martinez-Perez et al., 2012, Pedersen et al., 2016, Shiddiq et al., 2016, Tesi et al., 2016, Thiele et al., 2014, Yu et al., 2016, Zadrozny et al., 2017). Yet, the strong decoherence must be overcome to implement the envisaged application. Specific design criteria, such as nuclear-spin-free ligands (Yu et al., 2016), clock transitions (Zadrozny et al., 2017, Shiddiq et al., 2016), and low-energy vibrations (Atzori et al., 2017) have been developed to improve the quantum coherence time and temperature. As a matter of fact, the symmetry plays a vital part in spin-lattice relaxation process (namely, *T*_1_) (Ding et al., 2016). Consequently, it is very probable that the local symmetry of a spin carrier is equally important to the spin-spin relaxation process (namely, *T*_2_). However, the relationship between the decoherence and the local symmetries of spin carriers is still unclear.

Sessoli (Atzori et al., 2016a, Atzori et al., 2016b, Atzori et al., 2017, Tesi et al., 2016) and Freedman (Fataftah et al., 2016, Graham et al., 2014, Yu et al., 2016, Zadrozny et al., 2017) have contributed greatly to the development of molecular qubits with 3*d* transition metal ions as spin carriers. Nevertheless, up to now, only a few 4*f* metal centers showing the quantum coherence properties (Aguilà et al., 2014, Pedersen et al., 2016, Martinez-Perez et al., 2012, Thiele et al., 2014, Shiddiq et al., 2016) have been reported. Herein, we report two capped square antiprism neodymium complexes, [C(NH_2_)_3_]_5_[Nd(CO_3_)_4_H_2_O]∙2H_2_O (**1**) and [C(NH_2_)_3_]_4_[H_3_O][Nd(CO_3_)_4_H_2_O]∙9.5H_2_O (**2**), which have different local symmetries, *C*_1_ (**1**) versus *C*_4_ (**2**), in the neodymium moieties [Nd(CO_3_)_4_H_2_O]^5–^. Both neodymium-based complexes are easy-plane magnetic anisotropic and show field-induced slow magnetic relaxation behaviors, which is rare in lanthanide complexes. The quantum coherence phenomenon was observed by the 240 GHz pulsed EPR spectroscopies at low temperatures on undiluted complexes. Our work indicates that the higher symmetry results in the longer decoherence times, which is explained by the first principle calculations.

## Results and Discussions

### X-Ray Structural Studies

**Figure 1:**
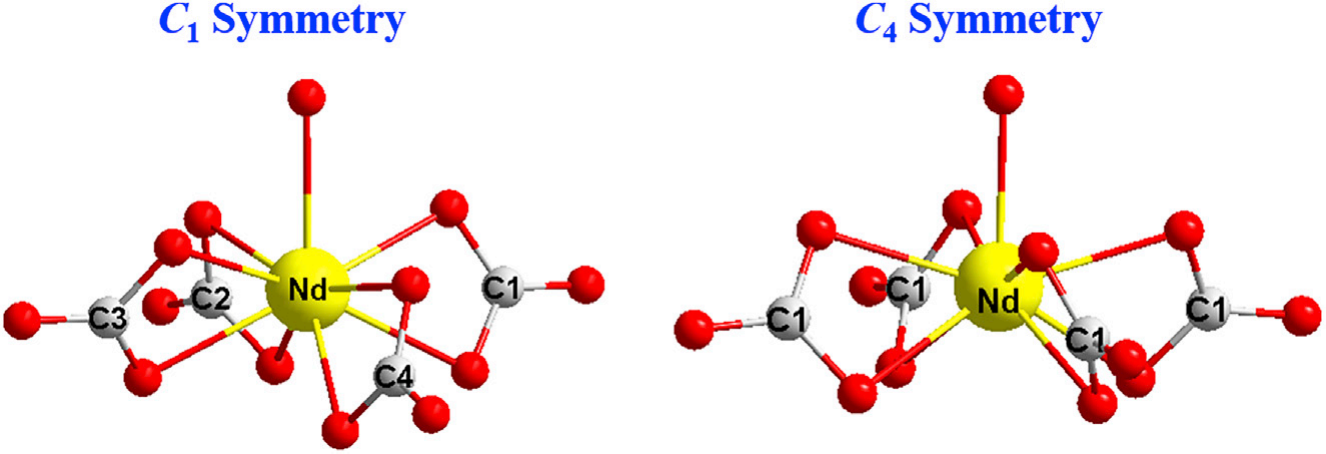
The Structures of 1 and 2 Crystallographically determined molecular structure of the [Nd(CO_3_)_4_(H_2_O)]^5−^ anion of **1** (left) and **2** (right). Neodymium, yellow; oxygen, red; carbon, gray. See also Figures S1, S2, S16, and S17.

Complexes **1** and **2** were synthesized according to a modified method reported with different rare-earth salts (Runde et al., 2000, Goff et al., 2010). The single crystal XRD reveals that **1** and **2** crystallize in the orthorhombic *P*na2_1_ and tetragonal *P*4/n space groups, respectively (Table S1). The [Nd(CO_3_)_4_H_2_O]^5–^ anion consists of four chelated CO_3_^2−^ anions and a bonded H_2_O molecule with a real *C*_1_ symmetry in **1** and *C*_4_ symmetry in **2** (Figure 1). For **1**, five guanidinium cations are arranged around the anion, forming a hydrogen-bonding network with two free water molecules in the crystal lattice (Figure S1), which makes the crystal stable in the air. For the lanthanide anion, the Nd(III) ion is in the plane of C1, C2, and C4 atoms (from carbonate) and the C3 atom is out of the plane with a mean deviation of 1.047 Å due to the steric hindrance. The related Nd-O lengths are in the range of 2.46–2.54 Å (Table S2). The coordinated water molecule is located on the quasi-*C*_4_ axis with a longest Nd-O length (2.63 Å). For **2**, four guanidinium and one H_3_O^+^ cations behave as charge-balanced ions, arranged around the anion, forming the hydrogen bonding with other ten water molecules in the crystal lattice, which is similar to the reported complex [C(NH_2_)_3_]_4_[H_3_O][Dy(CO_3_)_4_H_2_O]∙13H_2_O (Goff et al., 2010) as supported by thermogravimetric analysis (Figure S2). The crystal of **2** is unstable in the air at the room temperature but is quite stable below the ice point, which can be attributed to the large amount of lattice water molecules in the crystal lattice. In the lanthanide anion, the four C atoms from CO_3_^2−^ are in the same plane and the Nd(III) ion is out of the plane with a mean deviation of 0.335 Å. The lengths of Nd-O bonds are in the range of 2.477–2.508 Å. The coordinated water molecule is located on the *C*_4_ axis with a Nd-O length of 2.43 Å. More structural parameters are summarized in Table S2. If the carbonate anion is regarded as one coordination site, structure **2** could be considered to have the quasi-tetragonal pyramid symmetry with a *C*_4_ axis.

### Magnetic Properties

The direct-current (*dc*) magnetic susceptibilities of **1** and **2** were measured under 0.1 T in the temperature range of 1.8–300 K for **1** and 1.8–260 K for **2** (**2** is unstable above 260 K) (Figure S3). Notably, the *χ*_M_*T* value is 1.50 cm^3^mol^−1^K at 300 K for **1** (1.47 cm^3^mol^−1^K at 260 K for **2**), lower than the expected value of one isolated Nd(III) ion (1.64 cm^3^mol^−1^K, for *J* = 9/2, *g*_J_ = 8/11) (Wada et al., 2017). Given that the crystal of **1** is stable, regular, and big enough, magnetization measurements at low temperatures were collected along three different orientations (*a*, *b*, and *c*) to determinate the susceptibility tensor (Figure S4). Through the single-crystal XRD analysis, the magnetization is 1.44, 1.54, and 0.59 *Nμ*_B_ along the unit axes *a*, *c*, *b*, respectively. The magnetizations of *a* and *c* orientations increase quickly at low *dc* fields, slowly reaching to similar maximum values, whereas the magnetization of *b* orientation increases slowly in the whole field range, indicating that **1** is an “easy-plane” system. Treating the Nd(III) ion as an effective spin-1/2 ion, we could obtain the *g*-factor *g*_x_ = 2.97(1), *g*_y_ = 2.68(2), and *g*_z_ = 1.23(1) by fitting the magnetizations with the Brillouin function (Darby, 1967). Considering the similarity of local symmetries between **1** and **2**, their magnetic anisotropy might be similar. In order to confirm the “easy-plane” anisotropy of **1** and **2**, HF-EPR measurements on polycrystalline samples were conducted at 4.2 K and in the frequency range of 60–253 GHz (Figure S5) (Wang et al., 2012; Nojiri and Ouyang, 2012). From the HF-EPR spectra, the relevant *g*-factors were obtained as *g*_x_ = 3.00(2) and *g*_y_ = 2.56(2) for **1** and *g*_x_ = *g*_y_ = 2.79(2) for **2**, respectively, manifesting the “easy-plane” magnetic anisotropy for **1** and **2**. The resonance signals of *g*_z_ were not observed up to 22 T probably because the signals are too broad and hence weak due to the fast spin relaxations (Figure S6).

To study the origin of their magnetic properties, we performed the *ab initio* calculations (Karlstrom et al., 2003) for **1** and **2**. The results are summarized in Tables S6 and S7. The calculated magnetic axes of the ground state are shown in Figure S17. The calculated *g* values within CASSCF are *g*_x_ = 3.10(5), *g*_y_ = 3.01(7), and *g*_z_ = 0.86(8) for **1** and *g*_x_ = 3.12(1), *g*_y_ = 3.07(1), and *g*_z_ = 1.31(3) for **2**, which are in line with the aforementioned results from magnetization and HF-EPR measurements. The splitting of the two lowest Kramers doublets (KDs) for **1** was 99.5 cm^−1^ within CASSCF (128.6 cm^−1^ for **2**). Based on the observed *g* value of the lowest KDs, the ground state of **1** is mixed by several *m*_J_ states severely (Table S7), which may induce a large QTM between these states (proved by the high-field magnetization measurements as presented in Figure 3). The alternating-current (*ac*) magnetic susceptibility measurements were performed on **1** and **2** with polycrystalline samples at low temperatures. Without the external *dc* field, no out-of-phase susceptibility (*χ*_M_″) signal appeared as predicted by the *ab initio* calculations (Figures S7 and S10). This could be ascribed to the strong quantum tunneling of magnetization (QTM) at zero *dc* field, which is common in easy-plane-type systems as observed in the high-field magnetization measurements (Figure 3). When a small external *dc* field was applied, obvious signals in the frequency dependence of *χ*_M_″ were clearly observed in **1** and **2** (Figures S7 and S10), which might be due to the magnetic field-suppressed QTM or the strong phonon bottleneck effect. However, the maximum can only be observed in high frequency range under 1.5–2.0 kOe external *dc* field in **2**. This is the second time to discover that the light lanthanide complexes with the easy plane magnetic anisotropy can show slow spin relaxations (Table S8).

**Figure 2:**
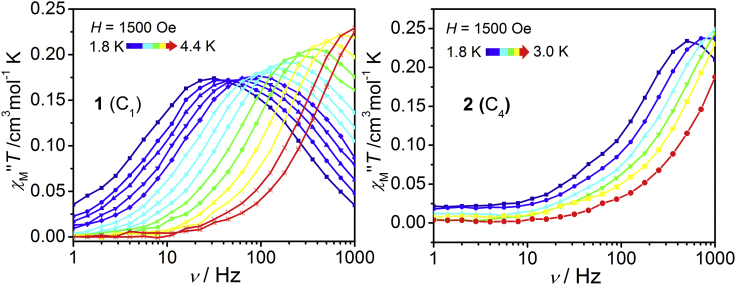
The Magnetic Relaxation of 1 and 2 Frequency dependence *ac* magnetic susceptibilities for **1** (left) and **2** (right) obtained under 1.5 kOe dc field. See also Figures S7–S14.

The temperature-dependent *ac* susceptibilities were measured under 1.5 kOe *dc* field (Figure 2, Figures S8 and S11). The relaxation times (*τ*) were obtained by fitting the Cole-Cole curves with the *CCFIT* program (Guo et al., 2011) (Figures S9 and S12). Complex **1** shows the slow magnetic relaxation in the temperature range of 1.8–4.4 K with relaxation times ranging from 4.39 ms at 1.8 K to 0.112 ms at 4.4 K. Complex **2** shows the slow magnetic relaxation in a lower temperature range (1.8–3.0 K), and the relaxation times (0.29–0.046 ms) are substantially lower than those of **1** (Figure S13). Complex **1** exhibits an exponential relationship for ln(*τ*) versus temperature, demonstrating that multiple relaxation mechanisms coexist in the relaxation process. By fitting the linear part in high temperature range (4.0–4.4 K for **1** and 2.4–3.0 K for **2**) with Arrhenius law *τ* = *τ*_0_ exp(–*U*_eff_/*k*_B_*T*), thermal energy barriers were obtained as *U*_eff_ = 30.7 K with *τ*_0_ = 1.05 × 10^−7^ s for **1** and *U*_eff_ = 9.25 K with *τ*_0_ = 2.09 × 10^−6^ s for **2**. The barriers are much smaller than the calculated energy splitting between ground state and the first excited state (Table S6). As a result, Raman process might dominate in the whole relaxation process, which is common in the easy-plane systems. Fitting of the complete temperature range data to a sum of direct and Raman processes with Equation 1:

(Equation 1) $$\tau^{-1}=AT+CT^{n}$$

affords *A* = 125.89 K^−1^s^−1^, *C* = 0.89K^−6.08^s^−1^, and *n* = 6.08 for **1** (*A* = 0, *C* = 413.85 K^−3.63^s^−1^, and *n* = 3.63 for **2**). Interestingly, the exponent *n* of 3.63 for **2** approaches the value of 3 as predicted in the case that both the acoustic and optical vibrations are important in the spin dynamic process. For **1**, the *n* of 6.08 is close to 9, indicating that the acoustic vibration is dominated in the relaxation process (Abragam and Bleaney, 2012).

Phonon-bottleneck effect (PB effect) usually plays a crucial part in the slow magnetic relaxation of “easy plane” system (Zadrozny et al., 2012). For a phonon-supported relaxation process, the energy exchange occurs through two processes (Abragam and Bleaney, 2012): from spin to phonon (*τ*_sp_) and from phonon to heat bath (*τ*_pb_). So the relaxation time (*τ*) can be described in Equation 2:

(Equation 2) $$\tau =\tau_{sp}+\frac{C_{s}}{C_{p}}\tau_{pb\;}$$

where *C*_s_ and *C*_p_ represent the heat capacity of spins and phonons in crystals, respectively. For an SMM, the phonon in crystal is abundant, so the rate-determining step in the relaxation process is *τ*_sp_. Hence, the relaxation time *τ* ≈ *τ*_sp_. The PB describes the situation that the number of spins is much larger than that of available phonons, in which the *C*_s_/*C*_p_ can be in the order of 10^4^–10^6^. The theoretical PB relaxation time can be calculated through the following Equation 3:

(Equation 3) $$\tau =\tau_{pb}\left(\frac{2\pi^{2}v^{3}N}{3\omega^{2}\Delta \omega }\right)tanh^{2}\left(\hbar \omega /2k_{B}T\right)$$

where the *τ*_ph_ is the mean lifetime of lattice phonon, *v* is the averaged sound speed in the crystal, *N* is the spin carrier density, and *ω* is the resonant frequency of vibration modes. For complexes **1** and **2**, the vibration modes would be nearly the same due to the similar chemical composition. If the magnetization relaxation phenomena of **1** and **2** were supported by PB effect, the relaxation time τ would be sensitive to the spin density. From the crystal parameters, the spin density is *N*_1_ = 0.00144 Nd/Å^3^ in **1** and *N*_2_ = 0.00111 Nd/Å^3^ in **2**. Here, *N*_1_/*N*_2_ > 1, *τ*_1_ would be longer than *τ*_2_ at the same temperature, which is in agreement with the experimental results. On the other hand, in the PB-dominated relaxation process, the diffusion to the crystal boundary should be taken into consideration. The relaxation time, *τ*, is proportional consideration to the crystal size, L_1_ or L_2_. When the crystal is ground to a smaller size (Figure S14), the relaxation time would be short.

To justify the magnetization dynamic mechanism, *ac* measurements were tested on a ground powder sample of **1** at 2 K (Figures S7 and S14). After grinding, the size of the crystals became smaller, thus affecting only the single phonon process (Scott and Jeffries, 1962, Pedersen et al., 2015), so that Raman and Orbach processes would not be influenced. However, the maximum of *χ*_M_″ for the ground powder sample shifts to the higher frequency range, indicating that the magnetic relaxation is sensitive to the low energy region of the phonon spectrum and/or the scattering of phonons on the crystal boundaries (Orendáč et al., 2016). Accordingly, the phonon bottleneck effect dominates the magnetic relaxation in the crystalline sample of **1**. Owing to the air instability, the *ac* susceptibility of the ground powder sample of **2** could not be explored.

**Figure 3:**
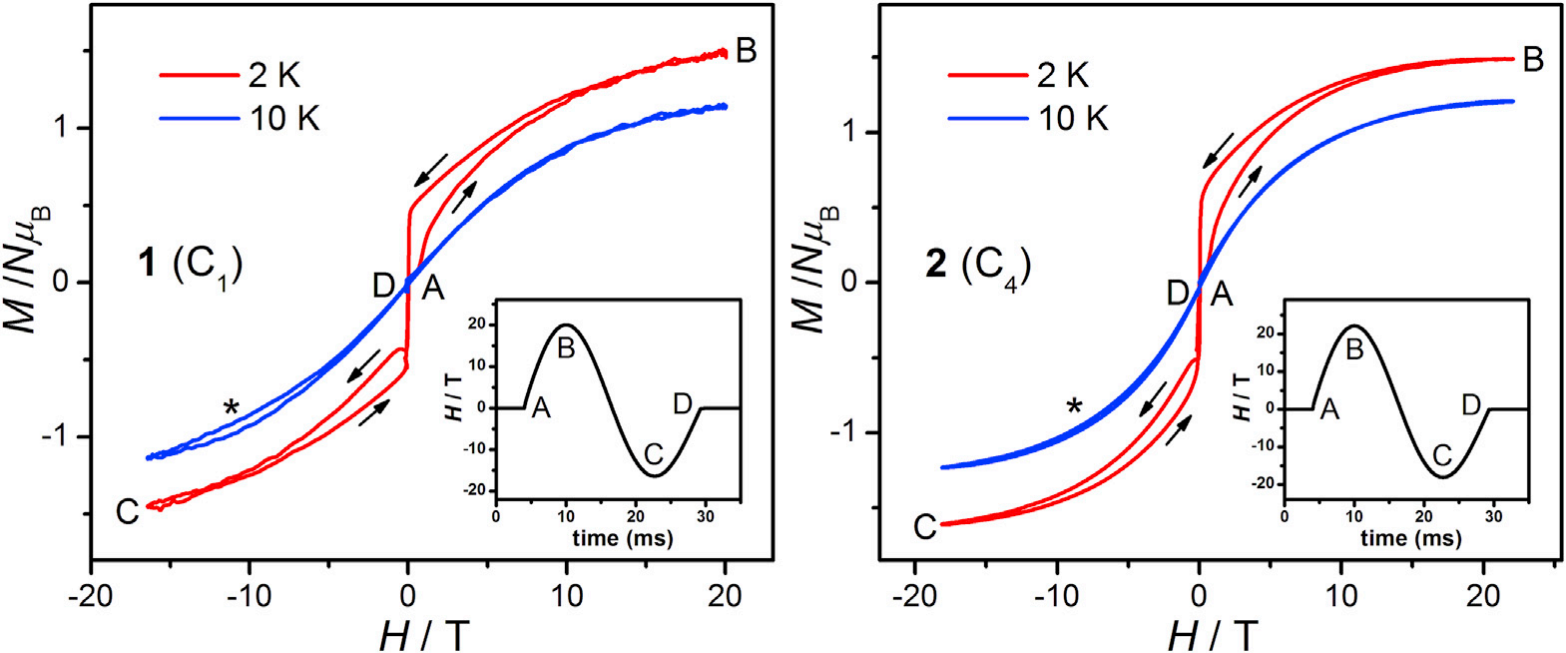
The Magnetic Hystersis Loop of **1** and **2** Magnetization versus pulsed magnetic field at 2 and 10 K for a powder sample of **1** (left) and **2** (right). The loop labeled by the asterisk is due to the experimental error when subtracting the background from the sample holder. Inset: Magnetic fields as a function of time. See also Figure S15.

The magnetic relaxation properties of **1** and **2** are further studied by the high-field magnetization measurements with a pulsed magnetic field (3000 T s^−1^ averagely, Figure 3) (Saito and Miyasata, 2001). At 2 K, by sweeping the pulsed field upwardly (A→B), the magnetization gradually increases to 1.5 *Nμ*_B_ at 20 T. In the down sweep (B→C), the magnetization decreases with a slower rate compared with upward sweeping, resulting in a pronounced hysteresis loop. A similar hysteresis loop is also observed in the negative field range. The hysteresis loops are due to the slow magnetic relaxations as observed in the *ac* susceptibility studies. The hysteresis loops were also tested at the same temperature using Squid VSM in a low field sweep rate (100 Oe/s) (Figure S15). No open loop was observed in this situation, which means that both **1** and **2** are not the magnet above 2 K. Thus, it is the pulsed magnetic field that makes it possible to observe the slow relaxation behaviors of **1** and **2** considering the extremely fast scan rate. Under the high sweep rate, the spin relaxation is in an adiabatic process, which limits the energy exchange between the phonons of the crystals and the environment (Lopez et al., 2009, Schenker et al., 2005). As a result, the butterfly loops were observed, indicating the strong phonon bottleneck effect in these complexes, which is in accordance with the *ac* susceptibility measurements.

However, it seems that phonon bottleneck effect mechanism has not been valued by chemists, although it might play an important role in many complexes with slow magnetic relaxation behaviors. For some special systems, for example, *S* = 1/2 systems like Cu(II) (Boča et al., 2017), isotropic systems like Gd(III) (Holmberg et al., 2015), also show slow spin relaxation under low temperature with external field. To explain the special slow spin relaxation in those systems, chemists often attribute the relaxation behavior to Raman process without further discussions, which deserve further studies. This work opens a way to discuss the nature of the slow spin relaxation behaviors, especially to understand the origin of the slow magnetic relaxation behaviors in *S* = 1/2 and isotropic systems.

### Quantum Coherence

**Figure 4:**
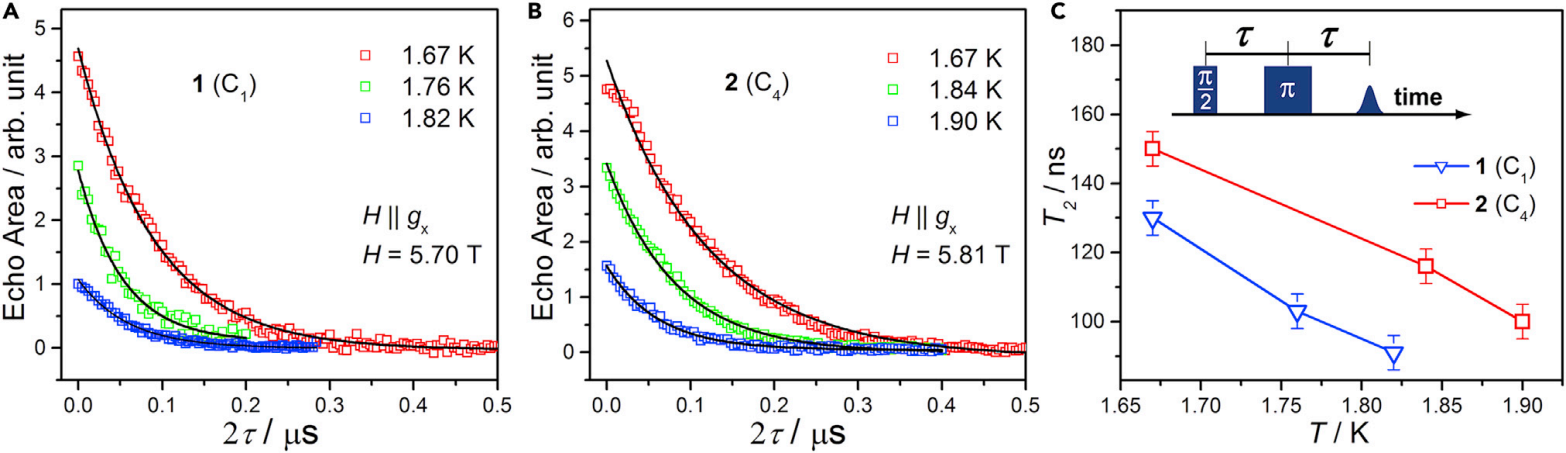
The Quantum Coherence of **1** and **2** (A and B) Echo signals as a function of 2*τ* at different temperatures and 240 GHz for **1** and **2**, respectively. Solid lines are the fits using a single exponential. (C) Temperature dependence of the spin-spin relaxation time, *T*_2_, for **1** and **2**.

Similar magnetization hysteresis loops were observed on a [Cu_3_] spin triangle complex whose life time is long enough to be detected by the pulsed EPR, proving [Cu_3_] complex a good candidate for qubit (Choi et al., 2012). So we studied the quantum decoherence properties of **1** and **2** on a 240 GHz pulsed EPR spectrometer at the National High Magnetic Field Laboratory, in Tallahassee, Florida, USA (van Tol et al., 2005, Morley et al., 2008). Measurements were done on single crystal samples, and the temperature dependence of quantum coherence time (*T*_2_) has been collected with the magnetic field along the *x* axes considering the easy-plane magnetic anisotropy for **1** and **2**. The spin coherence time was measured by a Hahn echo sequence (π/2 – *τ* – π – *τ* – echo), with the delay time *τ* varied during the measurements (Wernsdorfer et al., 2000; Schweiger and Jeschke, 2001). The widths of the pulses were tuned to maximize the echo signals and were typically between 100 and 150 ns. Figures 4A and 4B present the echo area as a function of 2*τ* at magnetic field 5.70 T for **1** and 5.81 T for **2** at different temperatures. The spin decoherence time *T*_2_ was extracted from the decay rate of the echo area, which was well fit by a single exponent function (exp(–2*τ*/*T*_2_)). Above 1.82 K for **1** (1.90 K for **2**), *T*_2_ becomes too short to give spin echoes with the limited time resolution of the pulsed spectrometer. Taking into account the measurement temperature range (1.67–1.82 K for **1** and 1.67–1.90 K for **2**), it is clear that the echo decays are strongly temperature dependent (Figure 4C) and *T*_2_ decreases from 130(5) ns at 1.67 K to 91(5) ns at 1.82 K for **1** (150(5) ns at 1.67 K to 100(5) ns at 1.90 K for **2**).

The spin decoherence time of **1** and **2** is close to that of the first single crystal SMM qubit [Fe_8_] (Takahashi et al., 2009). At the low temperature of 2 K and the strong magnetic fields (*H* = 5.70 T for **1** and 5.81 T for **2**), more than 99% of Nd spins are polarized to the lowest lying spin state, which suppresses the spin flip-flop process significantly. That is the most important reason why the echo can be observed under concentrated samples. The strong temperature dependence of *T*_2_ can be ascribed to spin bath (Takahashi et al., 2008) fluctuation dominated by an energy-conserving spin flip-flop process. To our knowledge, many factors like hyperfine coupling (Wernsdorfer et al., 2000), the distance of spin carriers, and so on, influence the decoherence path. Considering that more H_2_O molecules are around the [Nd(CO_3_)_4_H_2_O]^5–^ ion in **2** than in **1**, one would expect faster spin decoherence in **2**. However, the opposite was observed in our study, to put it more clearly, the spin decoherence time of **2** is longer than **1** at the same temperatures. According to the conclusion of Takahashi (Takahashi et al., 2011), the effect of nuclear spin and magnetic exciton on decoherence is much smaller than the phonon effect under high magnetic field and ultralow temperature. Here, the [Nd(CO_3_)_4_H_2_O]^5^^–^ cluster in **2** is *C*_4_ symmetric, whereas **1** is *C*_1_ symmetric. Therefore, the phonon spectrum of **1** would be more complicated than that of **2** because the local vibration modes in **1** are more than in **2**. The more complicated phonon freedom in **1** would increase the spin decoherence probability. From this point of view, the spin decoherence time of **2** with the high local symmetry should be longer at the same temperature and magnetic field.

### The First Principle Calculations

**Figure 5:**
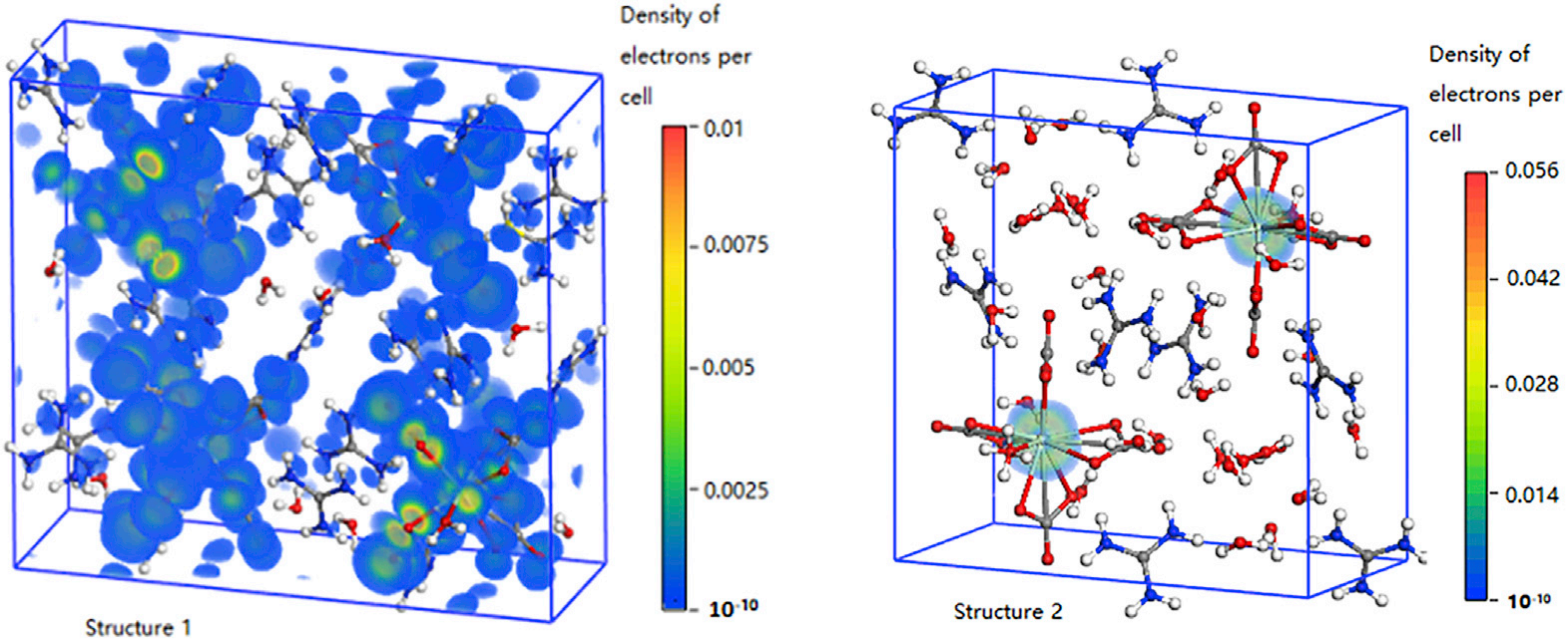
The Spatial Distributions of the Spin States of **1** and **2** The spatial distributions of the spin states at the Fermi level in Nd atom and its four ligands for both **1** and **2**. Green balls are for Nd atoms, red for O, blue for N, gray for C, and white for H. See also Figure S18.

To investigate the origin of the difference in the spin relaxation rate between **1** and **2**, we perform the first principle calculations for the eigenstates and eigenvalues of these two samples (Mendeley Data). It is found that, for both **1** and **2**, the magnetic moments mainly come from the *f* states of Nd atoms at the top of the valence band (Figure S18). The corresponding eigenstates, however, are very different for **1** and **2**. For **1**, the eigenstate at the top of the valence band is extended to both the Nd atom and the nearby four CO_3_^2−^ ligands, but for **2**, this eigenstate is localized within the Nd atom (Figure 5). This can be well understood from symmetry point of view. The Nd-O-C structure in **2** is *C*_4_ symmetric, so that the hopping channels of electrons between the Nd atom and its four nearby CO_3_^2−^ ligands can be suppressed most by the destructive interference, whereas such a suppression of hoppings does not happen in **1** as it has *C*_1_ symmetry. The different symmetry can lead to remarkable difference in the spin relaxation rate between **1** and **2**, because the spin-orbit interaction (SOI) together with phonons or charge fluctuations exert considerable influence in the spin relaxation processes (Khaetskii and Nazarov, 2001), and the hoppings between the Nd atom and its CO_3_^2−^ ligands contribute largely to the orbital motion of electrons in the spin state both in structures **1** and **2**. In this spin relaxation channel, the SOI provides the spin flip mechanism during the electron orbital motion, whereas the phonons or charge fluctuations cause the dissipation. The relaxation rate of the spin-flip process is proportional to the square of the absolute value of the spin-flip matrix element (Khaetskii and Nazarov, 2001), i.e. $\Gamma =\frac{1}{T_{1}}\propto {\left|\left(H^{↑↓}\right)\right|}^{2}$

In the present case, the main part of the orbital motion is due to the hopping processes between the Nd atoms and their ligands. Through the SOI, the spin-flip occurs during these hopping processes. Based on the above-mentioned results of the first principle calculations, the hopping processes between the Nd atom and its ligands are strongly suppressed in structure **2** due to the *C*_4_ symmetry, whereas in **1** such suppression does not happen. Therefore, in **2** the spin-flip processes due to the SOI are also suppressed, leading to the much longer spin-flip relaxation time in structure **2** compared with structure 1, *T*_1_(**2**) > *T*_1_(**1**). At the same time, *T*_2_ ≤ 2*T*_1_ (Golovach et al., 2004) is still valid for both structures **1** and **2**. It is also shown that, for localized spin states, such as the case in the quantum dots, one has *T*_2_ = 2*T*_1_ for all SOI mechanisms in leading order of the electron-phonon interaction (Golovach et al., 2004). The localization of the spin state in structure **2** is very similar to the case in the quantum dots, in which *T*_2_(**2**) = 2*T*_1_(**2**) holds. Hence *T*_2_(**2**) > *T*_2_(**1**) is reasonable as observed in our experiments.

## Conclusions

In summary, typical magnetic relaxation behaviors have been observed for two mononuclear Nd(III) complexes **1** and **2** with strong easy-plane magnetic anisotropy due to the strong phonon bottleneck effect. The spin decoherence studies reveal that the higher symmetry results in longer decoherence times, which is explained by the first principle calculations. Furthermore, consistent with the work of [GdW_10_] reported by Coronado (Martinez-Perez et al., 2012), we believe that the easy-plane magnetic anisotropy and high symmetry are extremely important factors to enhance spin decoherence time of molecular spin carriers. Further studies of spin decoherence in other lanthanide complexes are in progress in our laboratory.

### Limitations of the Study

This work demonstrates that the symmetry is an important factor to develop potential qubits with the improved performance and the higher symmetry results in longer decoherence times. This discovery provides a specific design criteria to develop potential qubits with improved performances. However, the decoherence times of complexes **1** and **2** are too short to application. And we need more couples of samples and more accurate physical model to understand the deep influence of symmetry in quantum coherence.

## Methods

All methods can be found in the accompanying Transparent Methods supplemental file.

## Data and Code Availability

Crystallographic data have been deposited in the Cambridge Crystallographic Data Centre under the accession numbers CCDC: 1546890 and 1860144.

## Supplemental Information

Document S1. Transparent Methods, Figures S1–S18, and Tables S1–S8

## References

[bib1] Abragam A., Bleaney B. (2012). Electron Paramagnetic Resonance of Transition Ions.

[bib2] Aguilà D., Barrios L.A., Velasco V., Roubeau O., Repollés A., Alonso P.J., Sesé J., Teat S.J., Luis F., Aromí G. (2014). Heterodimetallic [LnLn'] lanthanide complexes: toward a chemical design of two-qubit molecular spin quantum gates. J. Am. Chem. Soc..

[bib3] Atzori M., Morra E., Tesi L., Albino A., Chiesa M., Sorace L., Sessoli R. (2016). Quantum coherence times enhancement in vanadium(IV)-based potential molecular qubits: the key role of the vanadyl moiety. J. Am. Chem. Soc..

[bib5] Atzori M., Tesi L., Benci S., Lunghi A., Righini R., Taschin A., Torre R., Sorace L., Sessoli R. (2017). Spin dynamics and low energy vibrations: insights from vanadyl-based potential molecular qubits. J. Am. Chem. Soc..

[bib4] Atzori M., Tesi L., Morra E., Chiesa M., Sorace L., Sessoli R. (2016). Room-temperature quantum coherence and rabi oscillations in vanadyl phthalocyanine: toward multifunctional molecular spin qubits. J. Am. Chem. Soc..

[bib6] Boča R., Rajnák C., Titiš J., Valigura D. (2017). Field supported slow magnetic relaxation in a mononuclear Cu(II) complex. Inorg. Chem..

[bib7] Choi K.Y., Wang Z., Nojiri H., van Tol J., Kumar P., Lemmens P., Bassil B.S., Kortz U., Dalal N.S. (2012). Coherent manipulation of electron spins in the {Cu3} spin triangle complex impregnated in nanoporous silicon. Phys. Rev. Lett..

[bib8] Darby M.I. (1967). Tables of the Brillouin function and of the related function for the spontaneous magnetization. Br. J. Appl. Phys..

[bib9] Ding Y.-S., Chilton N.F., Winpenny R.E.P., Zheng Y.-Z. (2016). On approaching the limit of molecular magnetic anisotropy: a near-perfect pentagonal bipyramidal dysprosium(III) single-molecule magnet. Angew. Chem. Int. Ed..

[bib10] Fataftah M.S., Zadrozny J.M., Coste S.C., Graham M.J., Rogers D.M., Freedman D.E. (2016). Employing forbidden transitions as qubits in a nuclear spin-free chromium complex. J. Am. Chem. Soc..

[bib11] Goff G.S., Cisneros M.R., Kluk C., Williamson K., Scott B., Reilly S., Runde W. (2010). Synthesis and structural characterization of molecular Dy(III) and Er(III) tetra-carbonates. Inorg. Chem..

[bib12] Golovach V.N., Khaetskii A., Loss D. (2004). Phonon-induced decay of the electron spin in quantum dots. Phys. Rev. Lett..

[bib13] Graham M.J., Zadrozny J.M., Shiddiq M., Anderson J.S., Fataftah M.S., Hill S., Freedman D.E. (2014). Influence of electronic spin and spin-orbit coupling on decoherence in mononuclear transition metal complexes. J. Am. Chem. Soc..

[bib14] Guo Y.N., Xu G.F., Guo Y., Tang J. (2011). Relaxation dynamics of dysprosium(III) single molecule magnets. Dalton Trans..

[bib15] Holmberg R.J., Anh Ho L.T., Ungur L., Korobkov I., Chilbotaru L.F., Murugesu M. (2015). Observation of unusual slow-relaxation of the magnetization in a Gd-EDTA chelate. Dalton Trans..

[bib16] Karlstrom G., Lindh R., Malmqvist P.A., Roos B.O., Ryde U., Veryazov V., Widmark P.O., Cossi M., Schimmelpfennig B., Neogrady P., Seijo L. (2003). MOLCAS: a program package for computational chemistry. Comput. Mater. Sci..

[bib17] Khaetskii A.V., Nazarov Y.V. (2001). Spin-flip transitions between Zeeman sublevels in semiconductor quantum dots. Phys. Rev. B.

[bib18] Leuenberger M.N., Loss D. (2001). Quantum computing in molecular magnets. Nature.

[bib19] Lopez N., Prosvirin A.V., Zhao H., Wernsdorfer W., Dunbar K.R. (2009). Heterospin single-molecule magnets based on Terbium ions and TCNQF4 radicals: interplay between single-molecule magnet and phonon bottleneck phenomena investigated by dilution studies. Chem. Eur. J..

[bib20] Martinez-Perez M.J., Cardona-Serra S., Schlegel C., Moro F., Alonso P.J., Prima-Garcia H., Clemente-Juan J.M., Evangelisti M., Gaita-Arino A., Sese J. (2012). Gd-based single-ion magnets with tunable magnetic anisotropy: molecular design of spin qubits. Phys. Rev. Lett..

[bib21] Morley G.W., Brunel L.-C., van Tol J. (2008). A multifrequency high-field pulsed electron paramagnetic resonance/electron-nuclear double resonance spectrometer. Rev. Sci. Instrum..

[bib45] Nojiri H., Ouyang Z.W. (2012). THz Electron Spin Resonance on Nanomagnets. Terahertz Sci. Technol..

[bib22] Orendáč M., Tibenská K., Strečka J., Čisárová J., Tkáč V., Orendáčová A., Čižmár E., Prokleška J., Sechovský V. (2016). Cross-tunneling and phonon bottleneck effects in the relaxation phenomena of XY pyrochlore antiferromagnet Er2Ti2O7. Phys. Rev. B.

[bib23] Pedersen K.S., Dreiser J., Weihe H., Sibille R., Johannesen H.V., Sørensen M.A., Nielsen B.E., Sigrist M., Mutka H., Rols S. (2015). Design of single-molecule magnets: insufficiency of the anisotropy barrier as the sole criterion. Inorg. Chem..

[bib24] Pedersen K.S., Ariciu A.-M., McAdams S., Weihe H., Bendix J., Tuna F., Piligkos S. (2016). Toward molecular 4f single-ion magnet qubits. J. Am. Chem. Soc..

[bib25] Runde W., Neu M.P., Pelt C.V., Scott B.L. (2000). Single crystal and solution complex structure of Nd(CO_3_)_4_^5-^. The first characterization of a mononuclear lanthanide(III) carbonato complex. Inorg. Chem..

[bib26] Saito K., Miyasata S. (2001). Magnetic foehn effect in adiabatic transition. J. Phys. Soc. Jpn..

[bib27] Schenker R., Leuenberger M.N., Chaboussant G., Loss D., Güdel H. (2005). Phonon bottleneck effect leads to observation of quantum tunneling of the magnetization and butterfly hysteresis loops in (Et4N)3Fe2F9. Phys. Rev. B.

[bib28] Schweiger A., Jeschke G. (2001). Principles of Pulse Electron Paramagnetic Resonance.

[bib29] Scott P.L., Jeffries C.D. (1962). Spin-lattice relaxation in some rare-earth salts at Helium temperatures: observation of the phonon bottleneck. Phys. Rev..

[bib30] Sessoli R., Gatteschi D., Caneschi A., Novak M.A. (1993). Magnetic bistability in a metal-ion cluster. Nature.

[bib31] Shiddiq M., Komijani D., Duan Y., Gaita-Ariño A., Coronado E., Hill S. (2016). Enhancing coherence in molecular spin qubits via atomic clock transitions. Nature.

[bib32] Takahashi S., Hanson R., van Tol J., Sherwin M.S., Awschalom D.D. (2008). Quenching spin decoherence in diamond through spin bath polarization. Phys. Rev. Lett..

[bib33] Takahashi S., van Tol J., Beedle C.C., Hendrickson D.N., Brunel L.C., Sherwin M.S. (2009). Coherent manipulation and decoherence of S = 10 single-molecule magnets. Phys. Rev. Lett..

[bib34] Takahashi S., Tupitsyn I.S., van Tol J., Beedle C.C., Hendrickson D.N., Stamp P.C.E. (2011). Decoherence in crystals of quantum molecular magnets. Nature.

[bib35] Tesi L., Lucaccini E., Cimatti I., Perfetti M., Mannini M., Atzori M., Morra E., Chiesa M., Caneschi A., Sorace L., Sessoli R. (2016). Quantum coherence in a processable vanadyl complex: new tools for the search of molecular spin qubits. Chem. Sci..

[bib36] Thiele S., Balestro F., Ballou R., Klyatskaya S., Ruben M., Wernsdorfer W. (2014). Electrically driven nuclear spin resonance in single-molecule magnets. Science.

[bib37] van Tol J., Brunel L.-C., Wylde R.J. (2005). A quasioptical transient electron spin resonance spectrometer operating at 120 and 240 GHz. Rev. Sci. Instrum..

[bib38] Wada H., Ooka S., Yamamura T., Kajiwara T. (2017). Light lanthanide complexes with crown ether and its Aza derivative which show slow magnetic relaxation behaviors. Inorg. Chem..

[bib39] Wang Z., Datta S., Papatriantafyllopoulou C., Christou G., Dalal N.S., van Tol J., Hill S. (2011). Spin decoherence in an iron-based magnetic cluster. Polyhedron.

[bib44] Wang S.L., Li L., Ouyang Z.W., Xia Z.C., Xia N.M., Peng T., Zhang K.B. (2012). Development of high-magnetic-field, high-frequency electronic spin resonance system. Acta Phys. Sin..

[bib40] Wernsdorfer W., Caneschi A., Sessoli R., Gatteschi D., Cornia A., Villar V., Paulsen C. (2000). Effects of nuclear spins on the quantum relaxation of the magnetization for the molecular nanomagnet Fe8. Phys. Rev. Lett..

[bib41] Yu C.-J., Graham M.J., Zadrozny J.M., Niklas J., Krzyaniak M.D., Wasielewski M.R., Poluektov O.G., Freedman D.E. (2016). Long coherence times in nuclear spin-free vanadyl qubits. J. Am. Chem. Soc..

[bib42] Zadrozny J.M., Liu J., Piro N.A., Chang C.J., Hill S., Long J.R. (2012). Slow magnetic relaxation in a pseudotetrahedral cobalt(II)complex with easy-plane anisotropy. Chem. Commun. (Camb.).

[bib43] Zadrozny J.M., Gallagher A.T., Harris T.D., Freedman D.E. (2017). A porous Array of clock qubits. J. Am. Chem. Soc..

