# Supplementary material for: Controlling Electron Spin Decoherence in Nd-based Complexes via Symmetry Selection
Source: iScience. 2020 Feb 20;23(3):100926. doi: 10.1016/j.isci.2020.100926 (PMC7063258; doi:10.1016/j.isci.2020.100926)
Supplement: Document S1. Transparent Methods, Figures S1–S18, and Tables S1–S8 [file mmc1.pdf]

iScience, Volume *23*

Supplemental Information

Controlling Electron Spin Decoherence in Nd-based Complexes via Symmetry Selection

Jing Li, Lei Yin, Shi-Jie Xiong, Xing-Long Wu, Fei Yu, Zhong-Wen Ouyang, Zheng-Cai Xia, Yi-Quan Zhang, Johan van Tol, You Song, and Zhenxing Wang

Supporting Information

Supplemental Figures

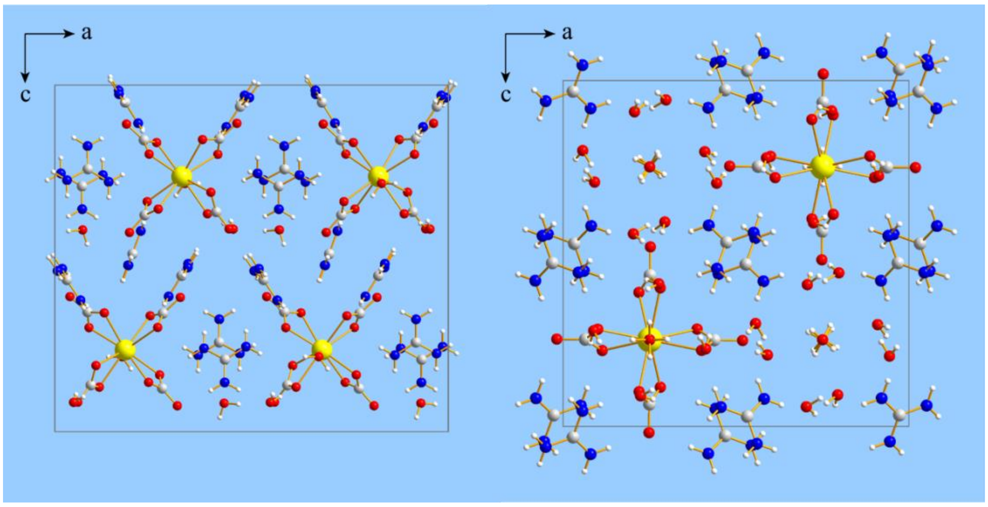

Figure S1. Packing diagrams of **1**(left) and **2** (right) in the crystal structure viewed along the *b* axis. Color codes: Nd, yellow; O, red; N, blue; C, gray; H, white. Related to Figure 1.

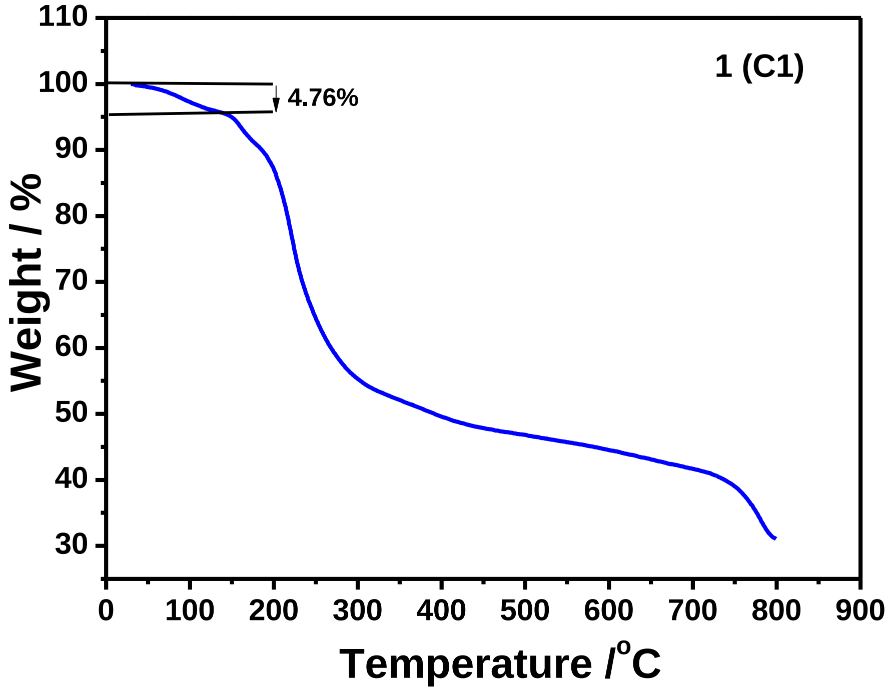

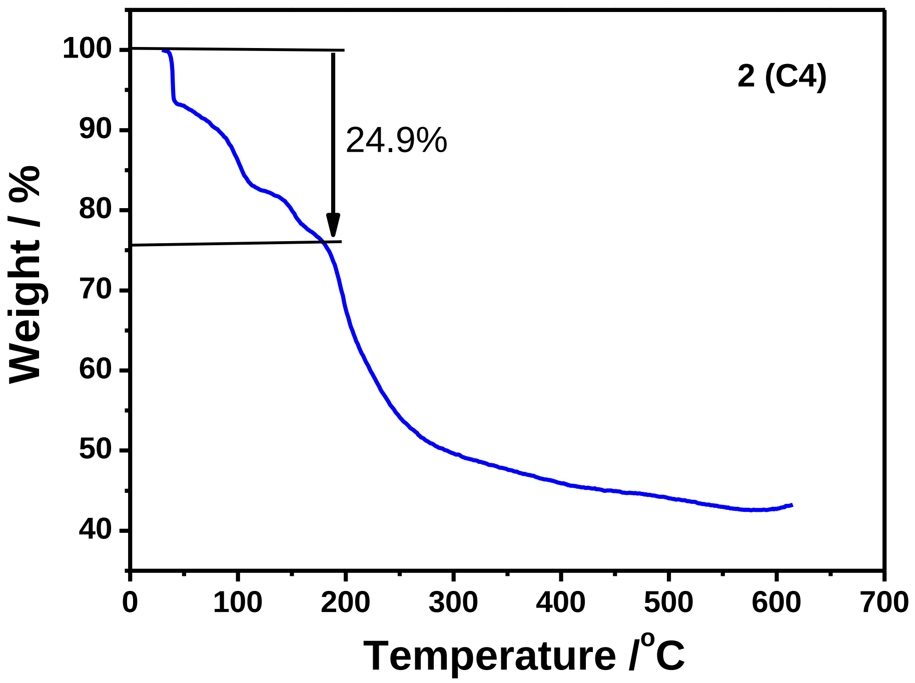

Figure S2. Thermogravimetric analysis of **1** (top) and **2** (bottom) in N_2. The ramp rates were 10 °C min^{-1} for **1** from 25 to 800 °C and 5 °C min^{-1} for **2** from 25 to 615 °C. Related to Figure 1.

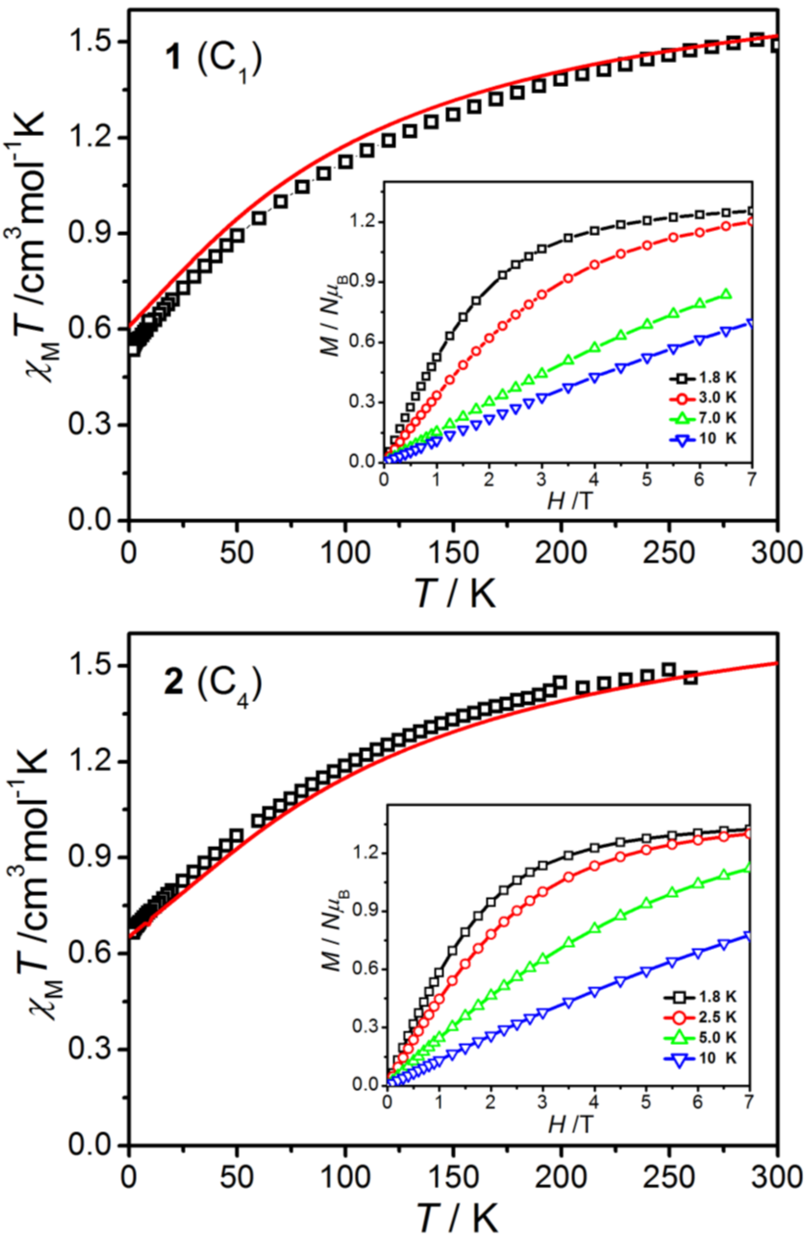

Figure S3. Temperature dependence of $\chi_M T$ under 1 kOe applied *dc* field at 1.8–300 K for a polycrystalline sample of **1**(top) and **2** (bottom) by MPMS-XL7. The solid line represents the calculated magnetic susceptibilities with CASSCF. Inset: Field dependence of magnetization under low temperature. Related to Figure 2.

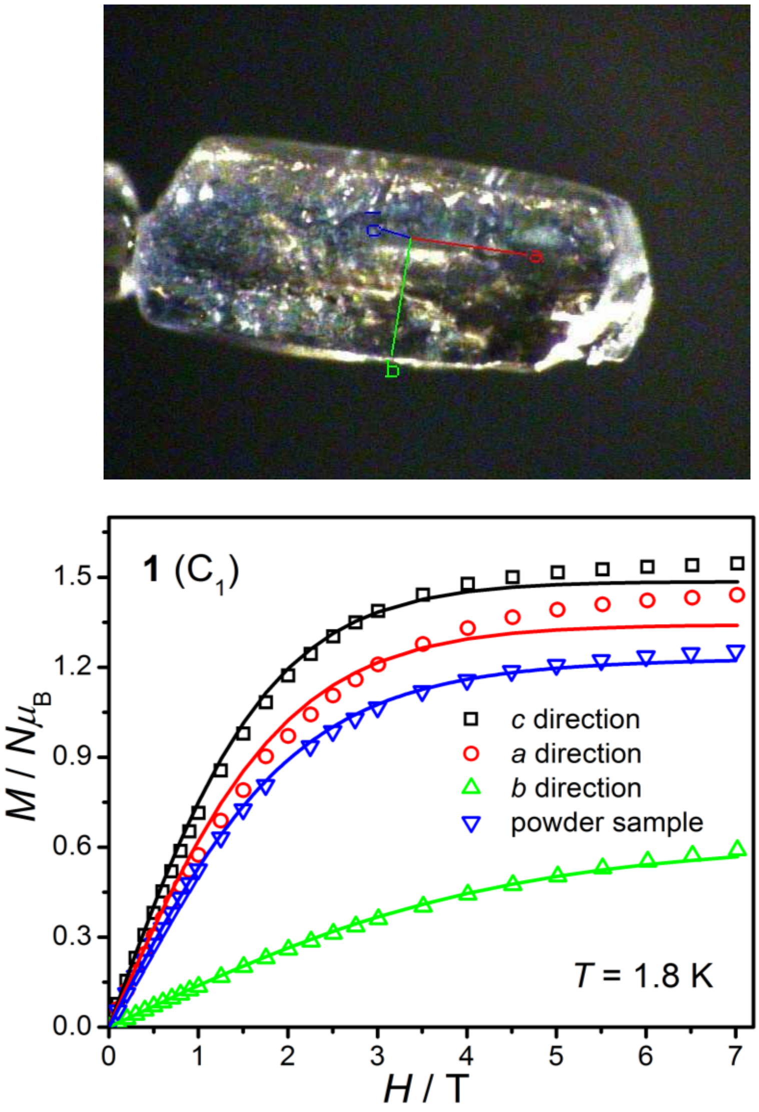

Figure S4. Top: The cell parameter *a*, *b*, *c* in the crystal from the single-crystal XRD analysis. Static magnetization data of a single crystal sample of **1**. Right: Low temperature magnetization *vs* *H*. the solid lines are best fits. Related to Figure 2.

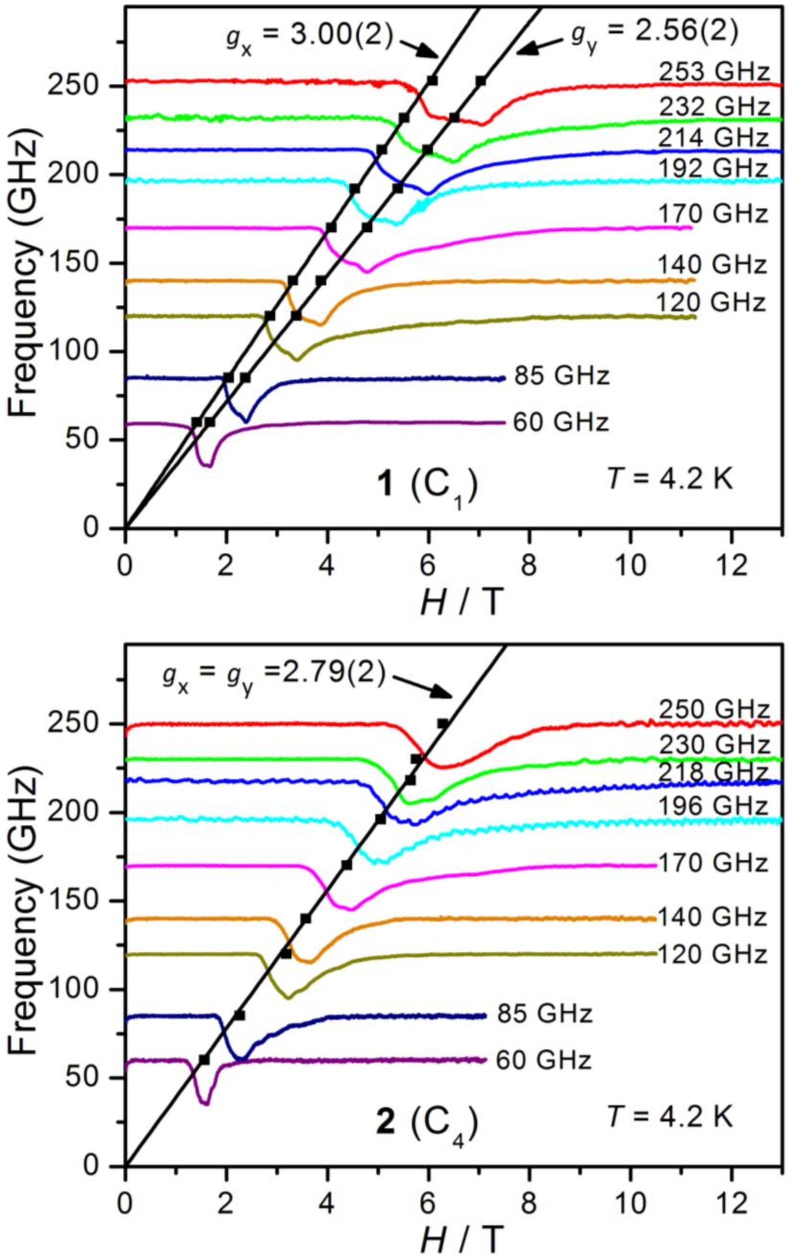

Figure S5. Variable-frequency EPR spectra collected on powder samples of **1** (top) and **2** (bottom) under 4.2 K. Solid lines are simulations of the frequency dependence of the peak positions employing the parameters given in the text. Related to Figure 2.

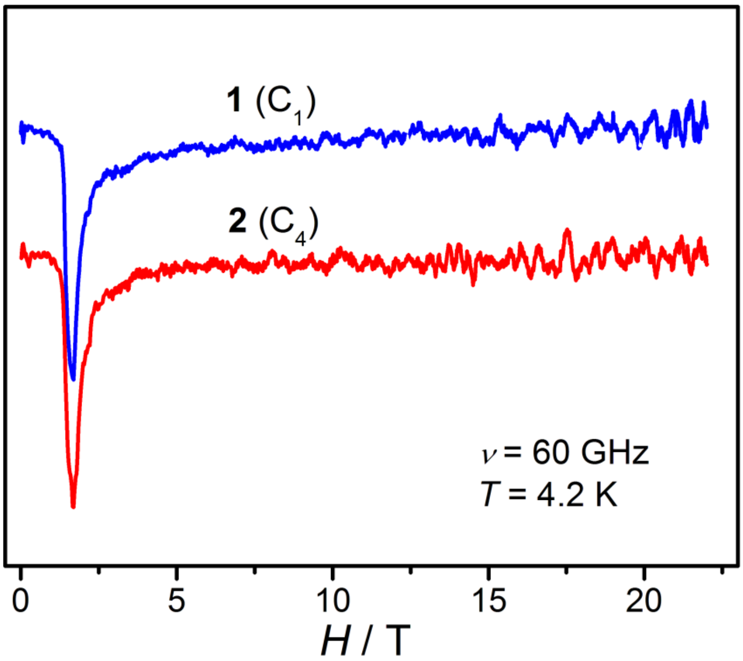

Figure S6. HF-EPR spectrum for a polycrystalline sample of **1** at 60 GHz and 4.2 K. Related to Figure 2.

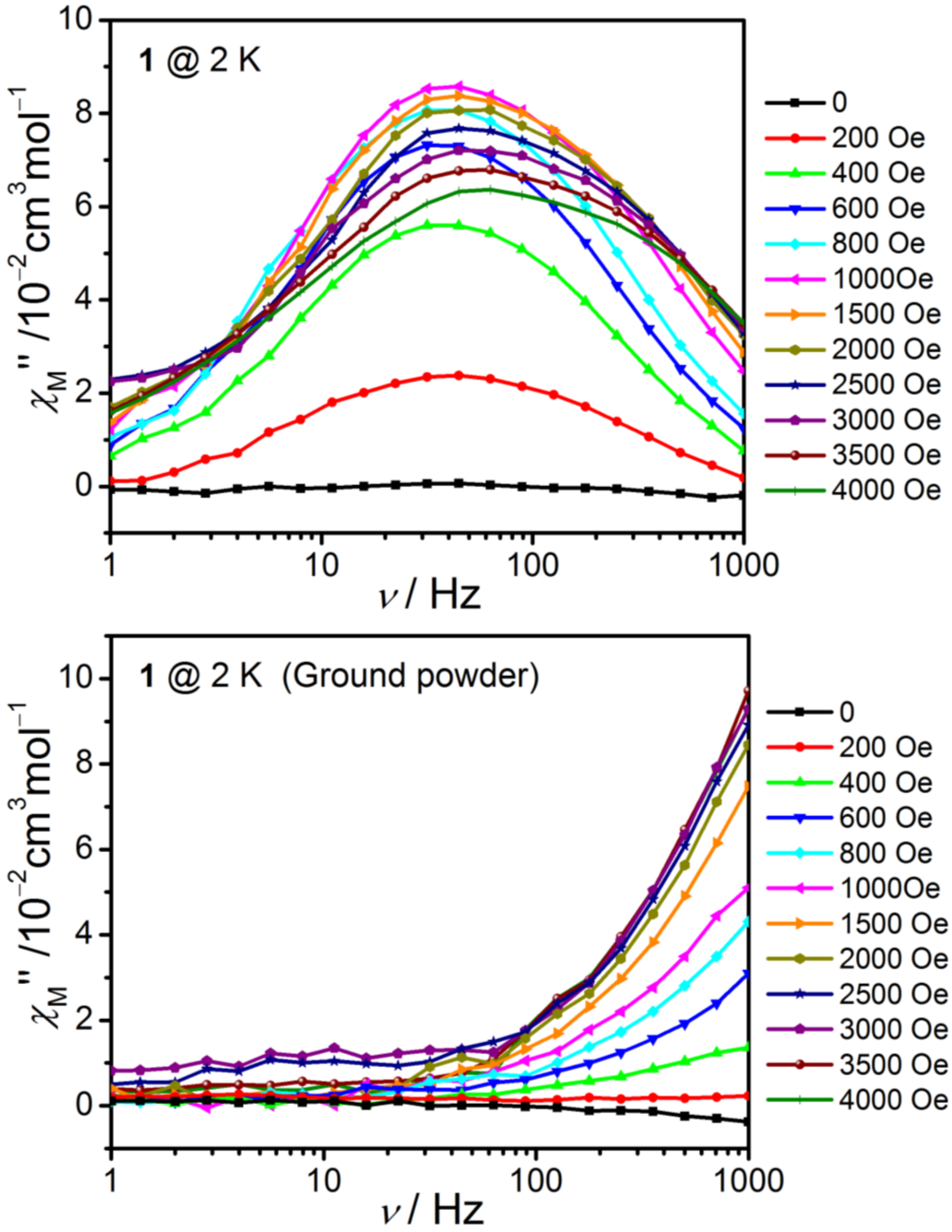

Figure S7. Variable-frequency out-of-phase χ_M'' components of the *ac* magnetic susceptibilities collected for a polycrystalline (top) and a ground (bottom) powder samples of **1** at 2.0 K under different applied *dc* fields. Related to Figure 2.

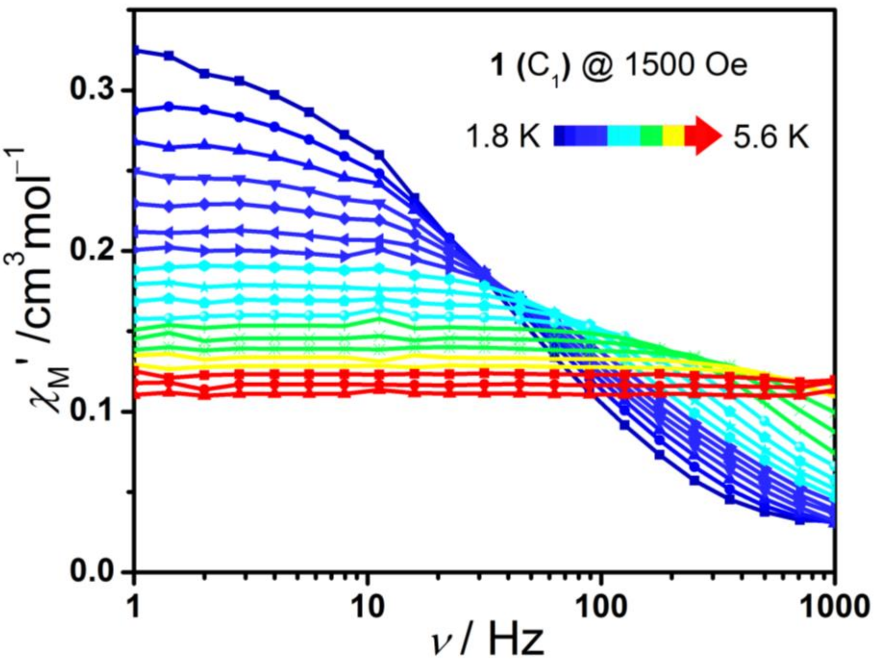

Figure S8. Frequency dependence of the in-phase (χ_M') *ac* susceptibilities under 1500 Oe *dc* field (1–999 Hz, by MPMS Squid VSM) at indicated temperatures for **1**. Related to Figure 2

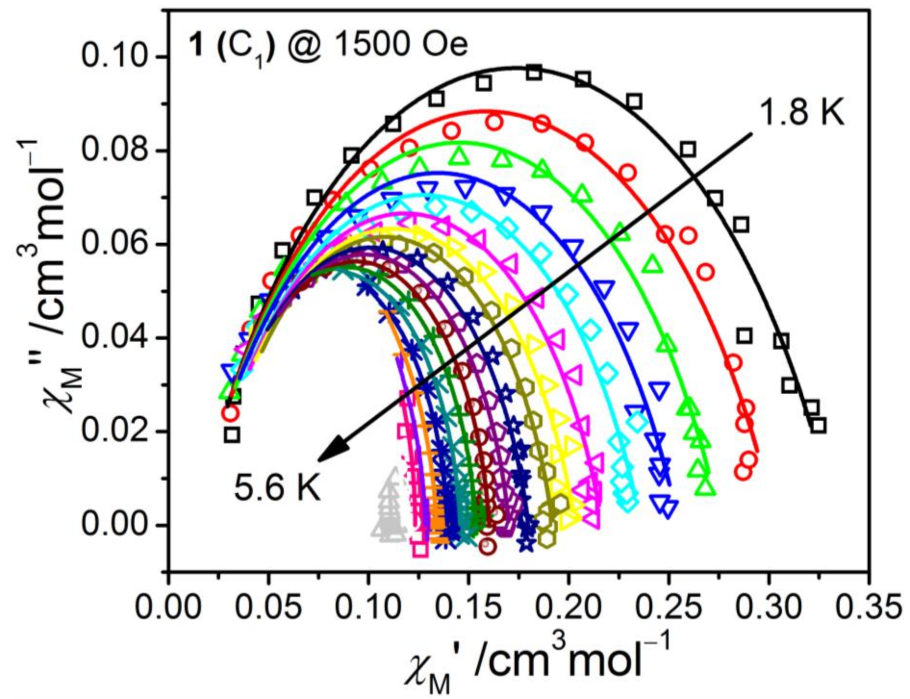

Figure S9. Variable temperature Cole-Cole plots under 1500 Oe *dc* field at different temperatures for complex **1**. Related to Figure 2

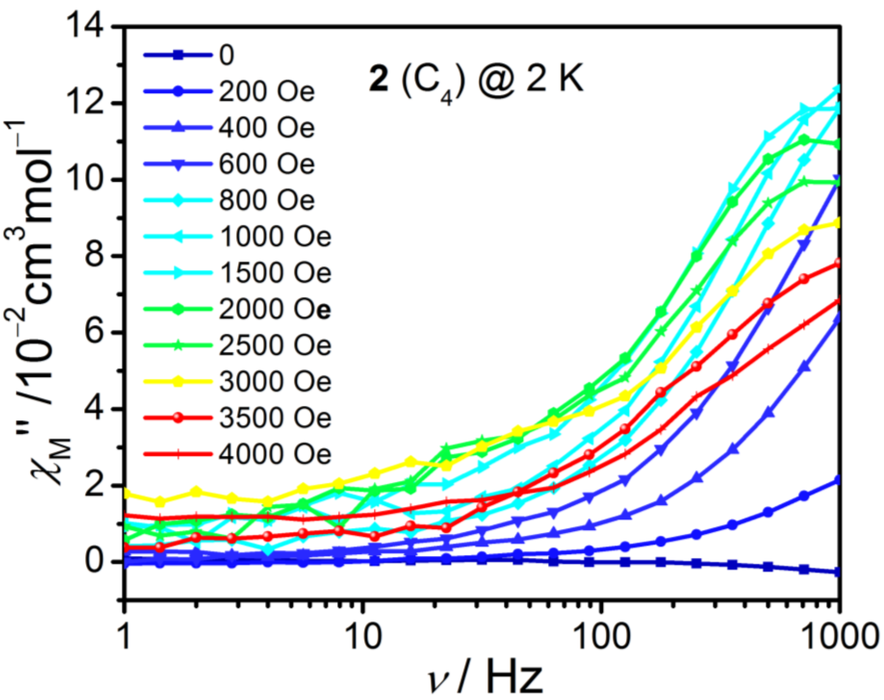

Figure S10. Variable-frequency out-of-phase χ_M'' components of the *ac* magnetic susceptibility collected for a polycrystalline sample of **2** at 2.0 K under different applied *dc* fields. Related to Figure 2.

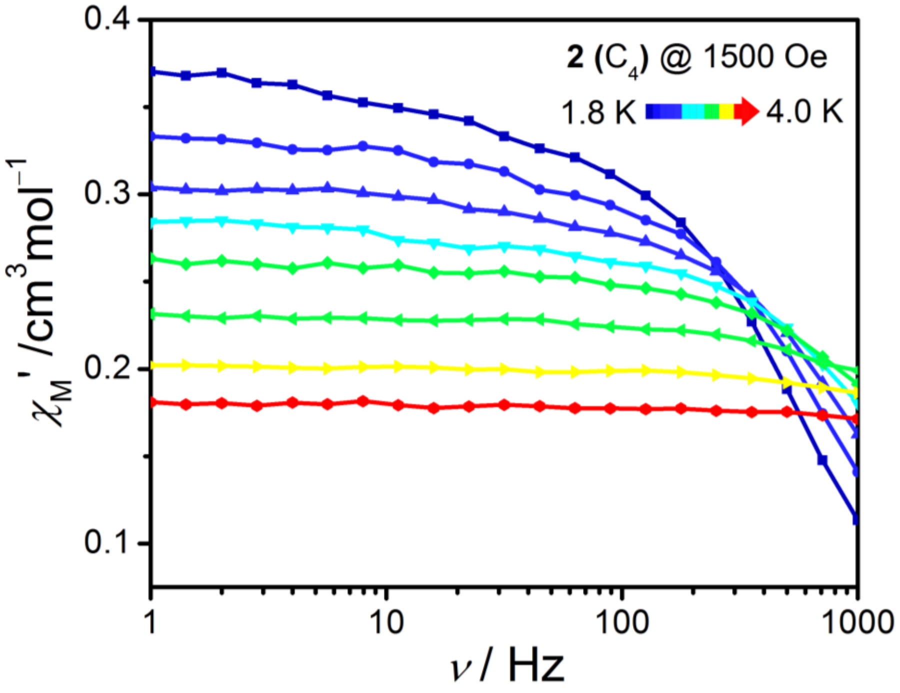

Figure S11. Frequency dependence of the in-phase (χ_M') *ac* susceptibilities under 1500 Oe *dc* field (1–999 Hz, by MPMS Squid VSM) at indicated temperatures for **2**. Related to Figure 2.

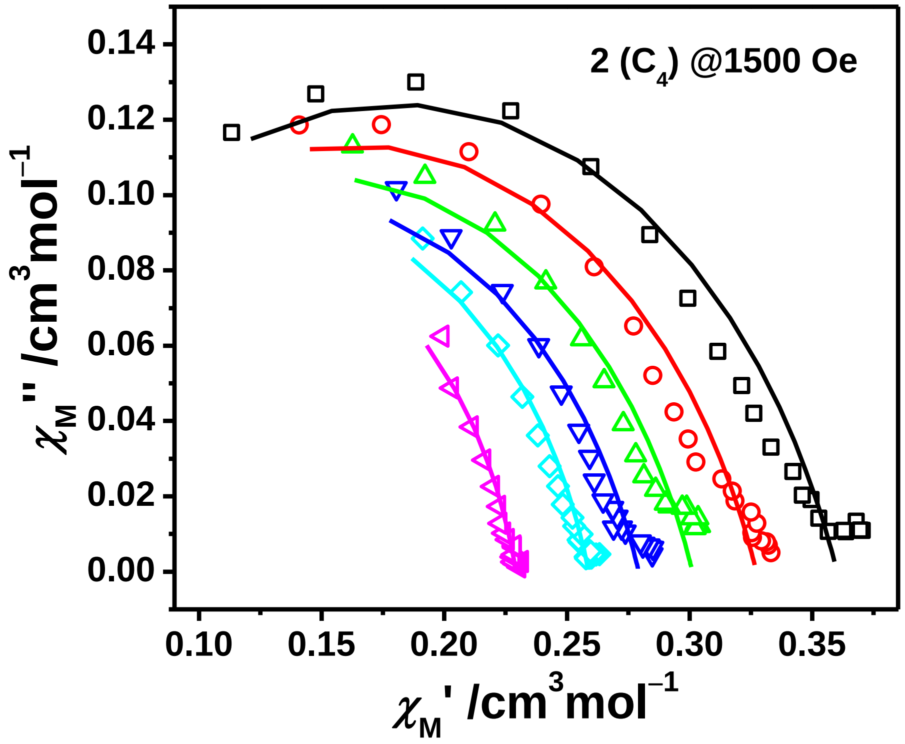

Figure S12. Variable temperature Cole-Cole plots under 1500 Oe *dc* field at indicated temperatures for complex **2**. Related to Figure 2.

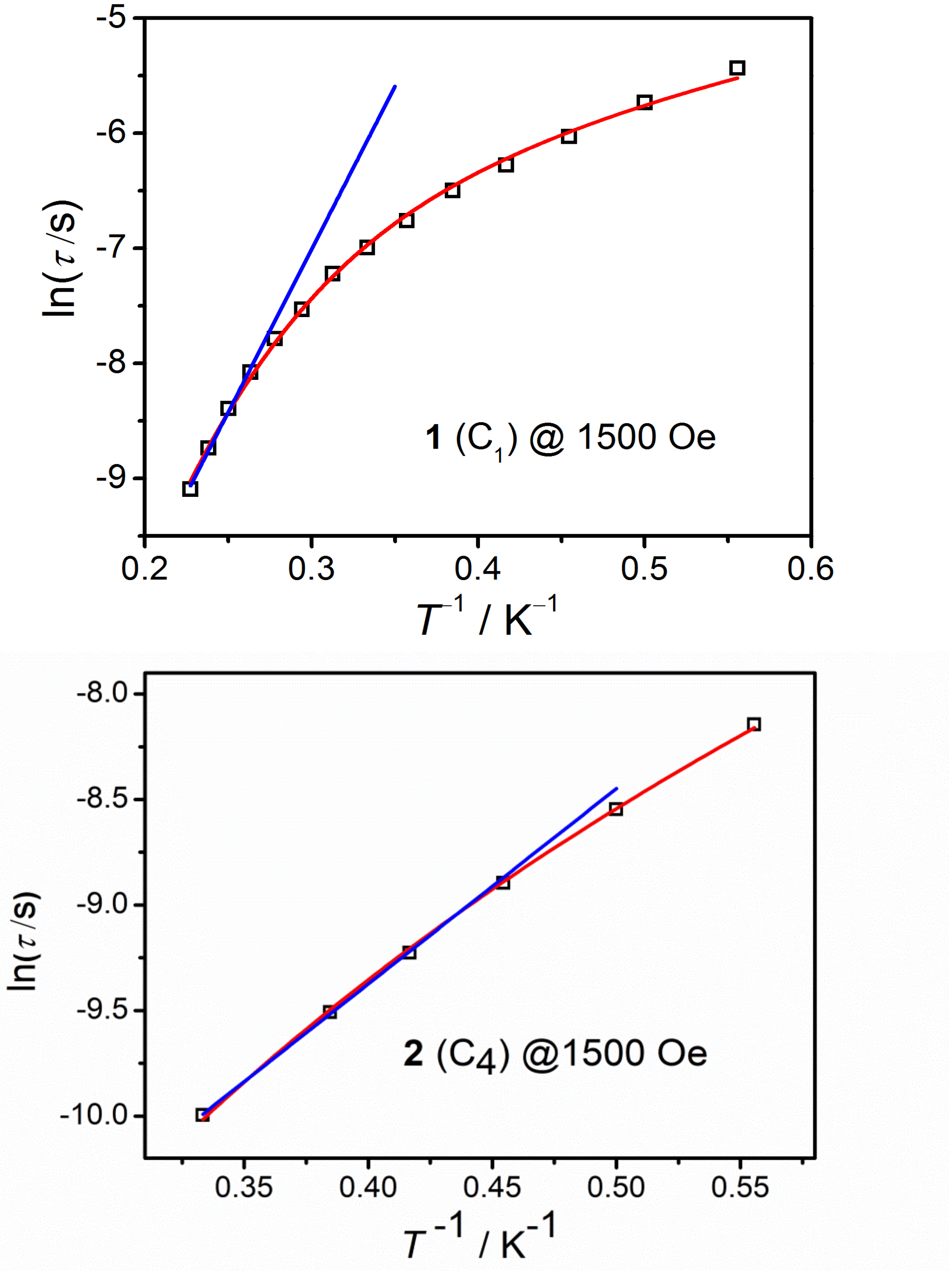

Figure S13. Arrhenius plot of ln(τ) as a function of T^{-1}. The blue solid lines are fitting results with Arrhenius law $\tau = \tau_0 \exp(U_{eff}/k_B T)$; red solid lines are fitting results with $\tau^{-1} = AT + CT^n$. Related to Figure 2.

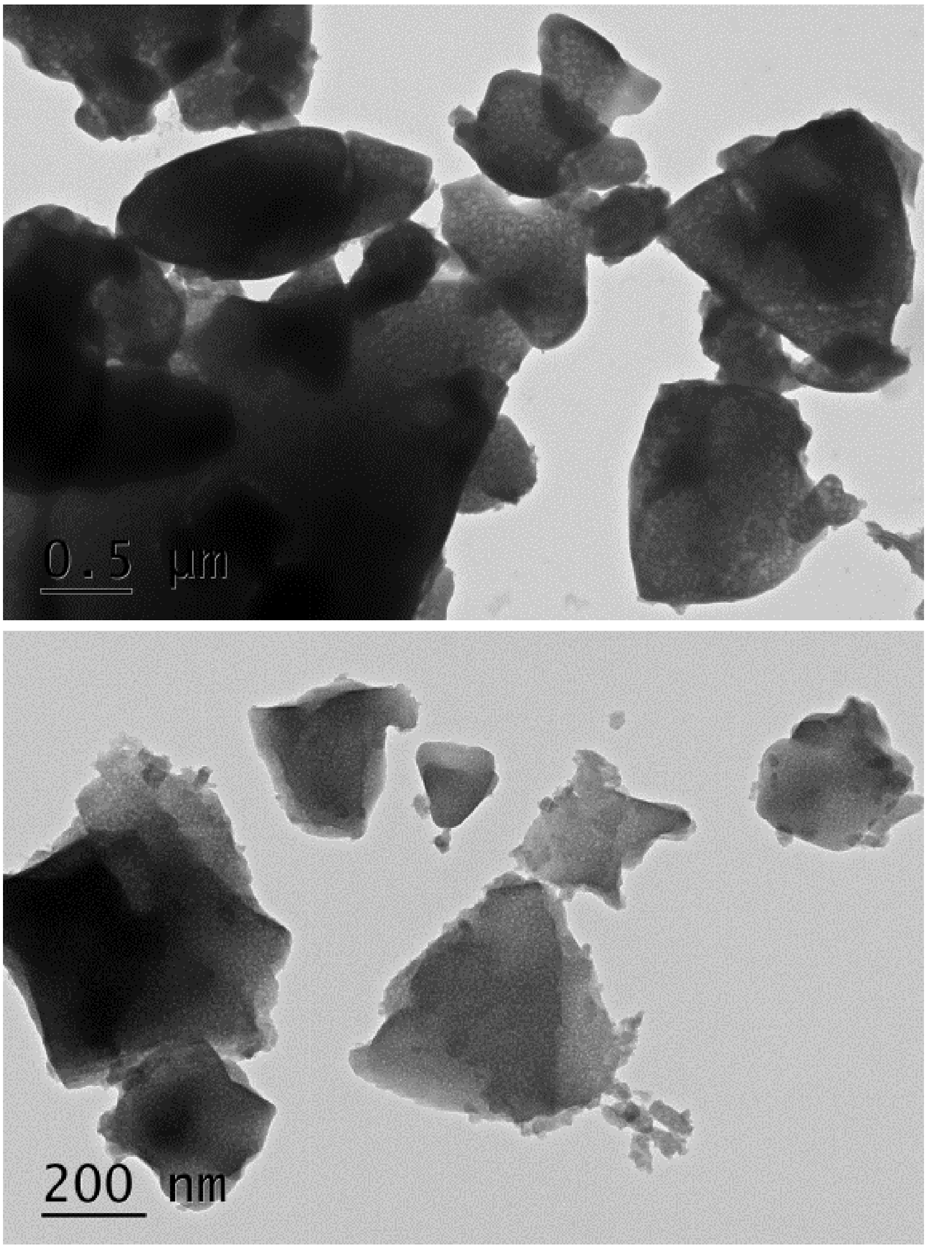

Figure S14. The SEM pictures of polycrystalline samples (Large crystals are too big to get the picture, and only same small ones are shown here. top) and ground samples (bottom). Related to Figure 2.

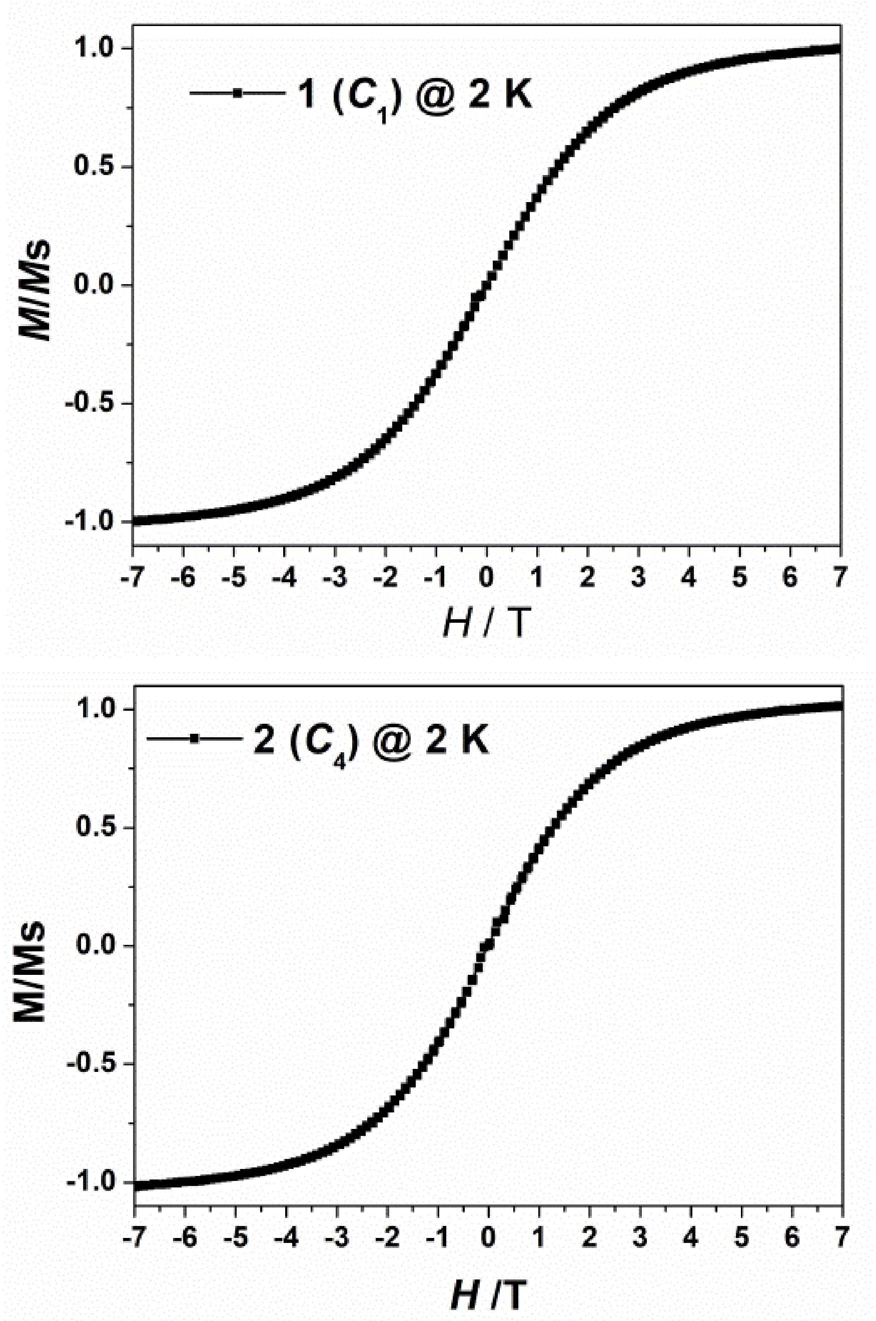

Figure S15. The magnetization curves for testing the magnetic hysteresis loops at 2 K using a VSM SQUID spectrometer by scanning the field at 100 Oe/s. Related to Figure 3.

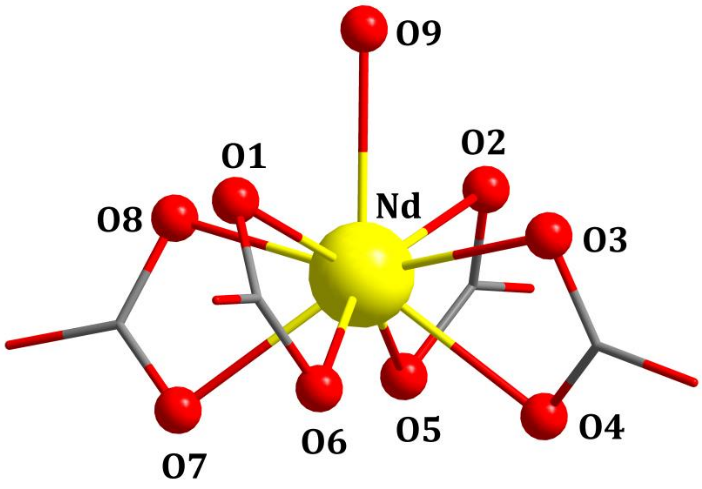

Figure S16. Calculated complete structures of complex **1** and **2**. H atoms are omitted. Related to Figure 1.

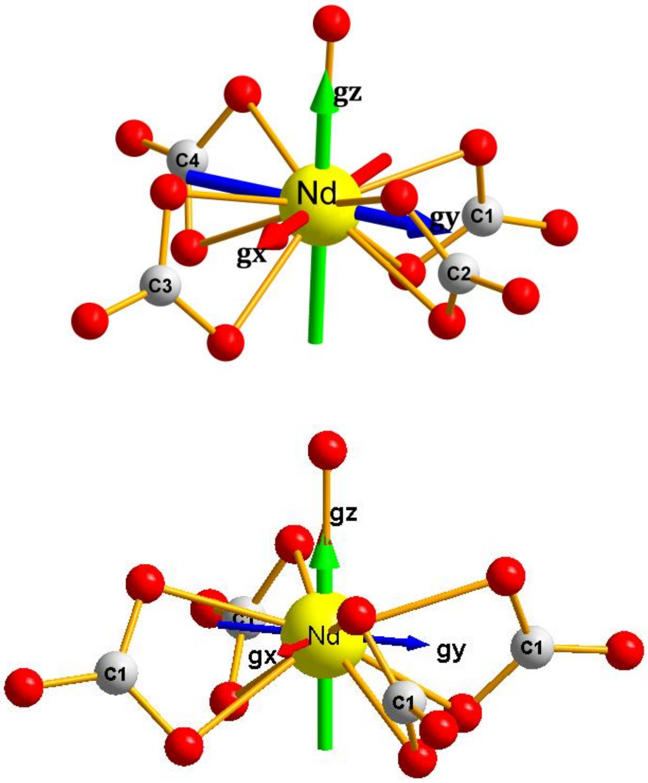

Figure S17. The calculated magnetic axes of complexes **1** (top) and **2** (bottom). Related to Figure 1.

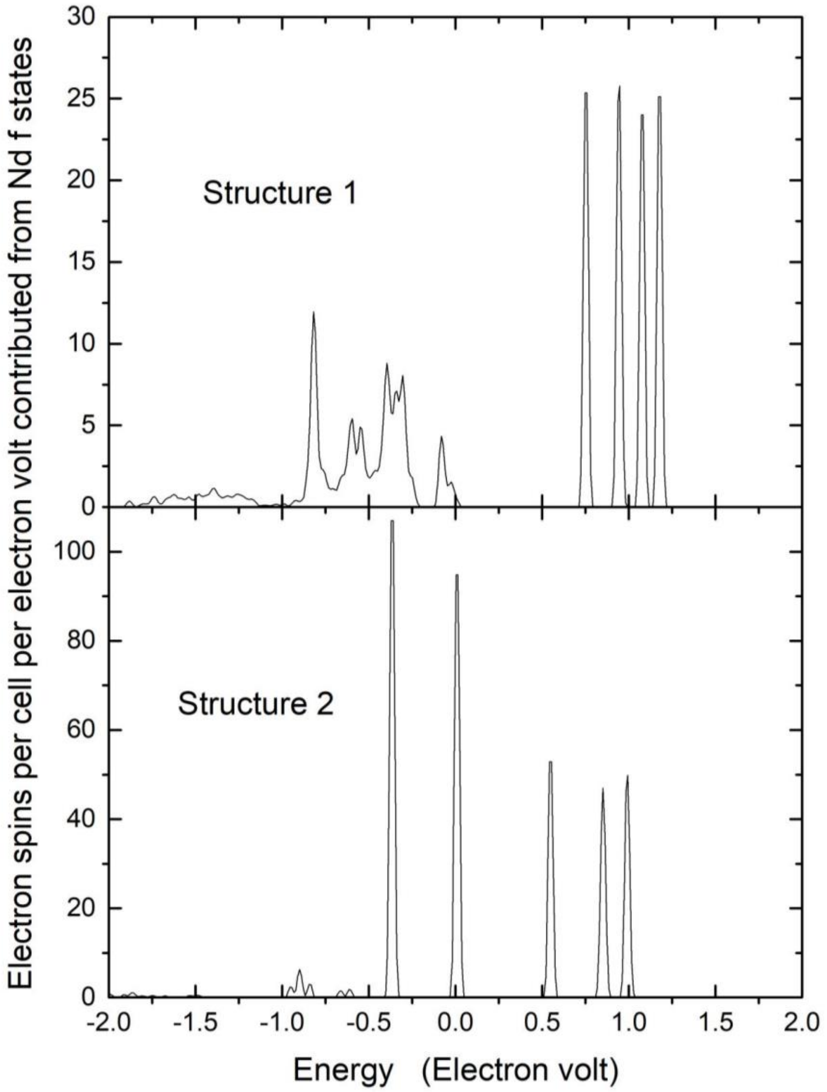

Figure S18. The calculated spins contributed from the *f* states of Nd ion as a function of energy for structures **1** and **2**. Related to Figure 5.

Supplemental tables

Table S1. Crystallographic data for complexes **1** and **2.**

	**1**	**2**
Formula	$C_9H_{36}N_{15}NdO_{15}$	$C_8H_{48}N_{12}NdO_{23.5}$
Mr[gmol^{-1}]	738.77	831.81
Crystal system	orthorhombic	tetragonal
Space group	$Pna2_1$	$P4/n$
a[Å]	21.438(4)	15.4630(9)
b[Å]	6.8275(15)	15.4630(9)
c[Å]	18.884(4)	7.5302(4)
α[º]	90.00	90
β[º]	90.00	90
γ[º]	90.00	90
V[Å^3]	2764.0(10)	1800.5(2)
T [K]	153	153
Z	4	2
ρ_{calcd} /g cm^{-3}	1.775	1.534
data measured	22530	14734
indep reflns	6776	9928
R_{int}	0.0516	0.0310
reflnswith I>2s(I)	6529	1953
parameter	363	140
Restraints	1	21
$R1$, $wR2$	0.0308 0.0767	0.0414 0.1155
GOF	1.065	1.193
CCDC	1546890	1860144

Table S2. Selected bond lengths (Å) and angles (°) for **1**. Related to Figure 1.

Nd-O1	2.542(4)	Nd-O2	2.466(4)
Nd-O4	2.489(4)	Nd-O5	2.470(4)
Nd-O7	2.511(4)	Nd-O8	2.462(4)
Nd-O10	2.483(4)	Nd-O11	2.455(4)
Nd-O13	2.622(5)		
O1-Nd-O2	52.53	O4-Nd-O5	53.31
O7-Nd-O8	52.94	O10-Nd-O11	53.14
O2-Nd-O13	71.2	O5-Nd-O13	77.31
O8-Nd-O13	68.54	O11-Nd-O13	78.64
C1-Nd-C2	89.90	C2-Nd-C3	86.51
C3-Nd-C4	89.75	C4-Nd-C1	90.59
C1-Nd-C3	172.49	C2-Nd-C4	154.01

Table S3. Selected bond lengths (Å) and angles (°) for **2**. Related to Figure 1.

Nd1-O1	2.507(3)	Nd1-O2	2.475(3)
Nd1-O4	2.431(7)		
O4-Nd1-O2	73.10(8)	O2-Nd1-O2^{i}	85.15(4)
O4-Nd1-O1	119.92(7)	O1-Nd1-O2	52.34(1)
O1-Nd1-O1^{i}	75.60(7)		

Table S4. Analysis of Cole-Cole plot of **1** under 1500 Oe *dc* field. Related to Figure 2.

T/K	χ_S	χ_t	T	α	R
1.8	1.39E-02	3.34E-01	4.39E-03	2.98E-01	4.36E-04
2.0	1.37E-02	3.03E-01	3.25E-03	2.98E-01	4.85E-04
2.2	1.48E-02	2.76E-01	2.41E-03	2.84E-01	4.48E-04
2.4	1.46E-02	2.55E-01	1.88E-03	2.83E-01	4.53E-04
2.6	1.80E-02	2.36E-01	1.51E-03	2.64E-01	4.53E-04
2.8	1.96E-02	2.17E-01	1.16E-03	2.39E-01	3.91E-04
3.0	2.11E-02	2.03E-01	9.20E-04	2.21E-01	2.94E-04
3.2	2.41E-02	1.92E-01	7.33E-04	1.88E-01	4.16E-04
3.4	2.20E-02	1.80E-01	5.37E-04	1.72E-01	3.08E-04
3.6	2.56E-02	1.70E-01	4.16E-04	1.33E-01	1.09E-04
3.8	2.77E-02	1.60E-01	3.12E-04	9.43E-02	1.55E-04
4.0	2.53E-02	1.54E-01	2.27E-04	8.79E-02	7.36E-05
4.2	2.42E-02	1.46E-01	1.61E-04	6.66E-02	1.02E-04
4.4	2.09E-02	1.39E-01	1.13E-04	5.49E-02	4.25E-05
4.6	5.10E-04	1.34E-01	6.67E-05	6.86E-02	6.69E-05
4.8	2.56E-15	1.28E-01	4.82E-05	4.59E-02	4.88E-05
5.0	5.01E-15	1.23E-01	3.71E-05	1.43E-03	8.19E-05
5.3	1.30E-14	1.17E-01	2.29E-05	1.43E-15	7.48E-05
5.6	2.13E-14	1.11E-01	1.43E-05	1.97E-15	6.49E-05

Table S5. Analysis of Cole-Cole plot of **2** under 1500 Oe *dc* field. Related to Figure 2.

T/K	χ_S	χ_t	T	α	R
1.8	5.54E-10	3.60E-01	2.91E-04	2.32E-01	1.48E-03
2	8.46E-10	3.27E-01	1.94E-04	2.30E-01	8.62E-04
2.2	1.13E-09	3.01E-01	1.37E-04	2.27E-01	1.24E-03
2.4	1.14E-09	2.79E-01	9.85E-05	2.19E-01	6.96E-04
2.6	1.67E-09	2.59E-01	7.43E-05	1.88E-01	2.57E-04
3	2.01E-09	2.29E-01	4.57E-05	1.54E-01	1.08E-04

Table S6. Calculated energy levels (cm^{-1}) and ***g*** (g_x, g_y, g_z) tensors of the lowest five Kramers doublets (KDs) of complex **1** and **2** calculated within CASSCF, respectively. Related to Figure 2.

KDs	**1**			**2**		
	CASSCF			CASSCF		
	E/cm^{-1}	***g***		E/cm^{-1}	***g***	
1	0.0	g_x	3.105	0.0	g_x	3.122
		g_y	3.017		g_y	3.071
		g_z	0.868		g_z	1.314
2	99.5	g_x	1.180	128.65	g_x	3.434
		g_y	1.500		g_y	2.964
		g_z	3.000		g_z	0.480
3	179.0	g_x	0.918	175.6	g_x	2.668
		g_y	1.217		g_y	2.636
		g_z	3.018		g_z	2.143
4	361.6	g_x	3.288	391.6	g_x	1.366
		g_y	2.111		g_y	1.423
		g_z	0.649		g_z	3.875
5	475.4	g_x	3.330	495.1	g_x	2.217
		g_y	2.548		g_y	2.419
		g_z	1.495		g_z	2.784

Table S7. In wave functions with definite projection of the total moment $|J_M>$ for complex **1** and **2** calculated within CASSCF and CASPT2, respectively. Related to Figure 2.

<table>
<tr><td rowspan="3">KDs</td><td colspan="2">1</td><td colspan="2">2</td></tr>
<tr><td colspan="2">CASSCF</td><td colspan="2">CASSCF</td></tr>
<tr><td>E/cm^{−1}</td><td>wave functions</td><td>E/cm^{−1}</td><td>wave functions</td></tr>
<tr><td>1</td><td>0.0</td><td>50%|±5/2>+46%|±3/2></td><td>0.0</td><td>57.36%|±5/2>+42.59%|±3/2></td></tr>
<tr><td>2</td><td>99.5</td><td>10%|±9/2>+34%|±5/2>+ 27%|±3/2>+28%|±1/2></td><td>128.65</td><td>42.23%|±5/2>+56.99%|±3/2> +</td></tr>
<tr><td>3</td><td>179.0</td><td>17%|±9/2>+9%|±7/2> +13%|±5/2>+21%|±3/2> +40%|±1/2></td><td>175.6</td><td>37.44%|±9/2>+60.29%|±1/2></td></tr>
<tr><td>4</td><td>361.6</td><td>15%|±9/2>+78%|±7/2></td><td>391.6</td><td>12.64%|±9/2>+84.74%|±7/2></td></tr>
<tr><td>5</td><td>475.4</td><td>57%|±9/2>+11%|±7/2> 30%|±1/2></td><td>495.1</td><td>49.54%|±9/2>+13.75%|±7/2> +36.68%|±1/2></td></tr>
</table>

Table S8. Literature survey for the SMMs based on light 4*f* elements. Related to Figure 2.

Metal ion	Formula	Anisotropy		E/k_B	τ_0 /s	H_{dc} /kOe	Refs.
		Easy axis	Easy plane				
Ce^{3+}	$Ce(NO_3)_3$(18-crown-6)[a]	√		30.3	2.20×10^{-7}	1.0	1
	$Ce(NO_3)_3$(1,10-diaza-18-crown-6)[a]	√		30.9	2.2×10^{-9}	1.0	1
	$[Ce(NO_3)\{Zn(L1)(SCN)\}_2]\cdot CH_3CN$	√		35.7	2.2×10^{-7}	1.0	2
	$[Ce(COT'')_2][Li(THF)_4]$		√	30	1.2×10^{-6}	0.4	3, 4
	$[Ce\{ZnI(L)\}_2(MeOH)]BPh_4\cdot 2MeOH\cdot$actone	√		21.2	1.6×10^{-7}	0	3,5
	$[Ce\{Zn(L)(AcO)\}_2]BPh_4$	√		37	2.7×10^{-7}	1.0	6
	$[Ce(dmso)_8][Ce(\eta^2\text{-}NO_3)_2(dmso)4(\alpha\text{-}Mo_8O_{26})_{0.5}][Mo_6O_{19}]$	-	-	24.4	2.56×10^{-7}	0.2	7
				4.4	2.09×10^{-5}		
				9.7	1.12×10^{-6}	1.4	
Nd^{3+}	$Nd(NO_3)_3$(18-crown-6)	√		45	2.6×10^{-8}	1.0	1
	$Nd(NO_3)_3$(1,10-diaza-18-crown-6)	√		73	1.4×10^{-10}	1.0	1
	$[Nd(NO_3)\{Zn(L1)(SCN)\}_2]\cdot CH_3CN$	√		38.5	2.07×10^{-7}	1.0	2
	$[L_2Nd(H_2O)_5][I]_3\cdot L_2\cdot(H_2O)$ (L=tBuPO(NHi-Pr)$_2$)	√		24.69	5.03×10^{-6}	0	8
				16.08	2.64×10^{-4}	0	
				39.21	8.98×10^{-7}	2.0	
	$Na_9[Nd(W_5O_{18})_2]\cdot 32H_2O$	√		74.1	3.55×10^{-10}	1.0	9
	$[Nd(COT'')_2][Li(THF)_4]$	-	-	21	5.5×10^{-5}	1.0	10
	$[C(NH_2)_3]_5[Nd(CO_3)_4(H_2O)]\cdot 2H_2O$		√	30.7	1.05×10^{-7}	1.5	**This work**
	$[C(NH_2)_3]_4[Nd(CO_3)_4H_2O]\cdot H_3O\cdot 13H_2O$		√	7.7	3.97×10^{-6}	1.5	**This work**

Transparent Methods

(1).Experimental section

Complex **1**: Guanidine carbonate (4.5 g) was dissolved in 10 mL of distilled water. To this was added $Nd(NO_3)_3 \cdot 6H_2O$ (0.5 g) in 5mL distilled water. Precipitate formed, and ten minutes later it was filtered off. The resulting clear solution was stored in refrigerator and the block crystals were obtained after three days. Elemental analysis: found (calcd for Nd4C36O60H144N60)/%: C 14.63 (14.64), H 4.91 (4.76), N 28.44 (28.35). IR (cm-1, KBr): 3500(vs), 3116(vs), 2815(m), 2352(w), 2242(w), 1682(vs), 1564(s), 1461(s), 1373(vs), 1163(m), 868 (s), 757 (m), 711(m), 558(m)

Complex **2**: Guanidine carbonate (4.5 g) was dissolved in 10 mL of distilled water. To this was added $Nd(SO_3CF_3)_3$ (1 g) in 5mL distilled water. Precipitate formed, and ten minutes later it was filtered off. The resulting clear solution was stored in refrigerator and the block crystals were obtained after a week. The block crystal can stable in the room temperature for 5-10 min, but it is stable during several weeks below 270 K.

(2).Physical measurements.

The IR spectra were carried out using a Nexus 870 FT-IR spectrometer with KBr pellets in the range from 500 to 4000 cm−1. Elemental analyses of C, N, H were measured on a Perkin Elmer 240C elemental analyzer. Thermogravimetric analyses (TGA) were performed on a STA449F3 TG-DSC instrument in flowing N2 at a heating rate of 5 °C per minute in the range of 25 to 600 °C. The static magnetic measurements were collected on MPMS-XL7 SQUID magnetometer. The alternating-current (ac) susceptibility measurements were collected on a Quantum Design VSM SQUID magnetometer. The static magnetic measurements were performed in the temperature range 1.8-300 K in a field of 1000 Oe and the magnetization isothermal measurements were performed in fields of between 0 and 7 T on a polycrystalline sample. The alternating-current (ac) susceptibility measurements were carried out under an oscillating field of 2 Oe with frequency ranging from 1 to 999 Hz. Experimental susceptibilities were corrected for diamagnetism using Pascal's constants and for the sample holder by previous calibration. Pulsed high-field magnetization and HF-EPR measurements were performed on locally developed instruments at the Wuhan National High Magnetic Field Center in China. Pulsed HF-EPR measurements were done on a super-heterodyne EPR spectrometer at the National High Magnetic Field Laboratory in USA. The raw spectra obtained in an absorptive mode were subsequently digitally transformed into a derivative presentation.

(3).Crystallographic data collection and refinement.

Crystallographic data of complexes **1** and **2** were collected on Bruker APEX-II CCD area-detector diffractometer with Mo-Kα radiation (λ = 0.71073 Å) using an φ and ω scans at low temperature. The diffraction data were integrated using SAINT,[11a] and were corrected for absorption using SADABS.[11b] All non-hydrogen atoms were located by the Patterson method.[11c] The structures were solved by direct methods and refined using the full-matrix least-squares technique within the SHELXTL program package.[11d] All non-hydrogen atoms

were refined with anisotropic displacement parameters. The hydrogen atoms were generated geometrically using the riding-model.

(4).Theoretical calculations mothed

Complete active space second-order multiconfigurational perturbation theory (CASPT2) considering the effect of the dynamic electron correlation based on complete-active-space self-consistent field (CASSCF) method with MOLCAS 8.0 program package was performed on Nd^{3+} fragment (see Figure S15 for the calculated complete structure) on the basis of X-ray determined geometry of complex **1** and **2**. For CASSCF calculations, the basis sets for all atoms are atomic natural orbitals from the MOLCAS ANO-RCC library: ANO-RCC-VTZP for Nd^{3+} ion; VTZ for close O; VDZ for distant atoms. The calculations employed the second order Douglas-Kroll-Hess Hamiltonian, where scalar relativistic contractions were taken into account in the basis set. The effect of the dynamical electronic correlation was applied using CASPT2 based on the first CASSCF calculation. After that, the spin-orbit coupling was handled separately in the restricted active space state interaction (RASSI-SO) procedure. The active electronsin 7 active spaces include all *f* electrons (CAS(3 in 7) for complex **1**)in the CASSCF calculation. To exclude all the doubts we calculated all the roots in the active space. We have mixed the maximum number of spin-free state which was possible with our hardware (all from 35 sextets and 80 from 112 doublets).

In order to investigate the origin of the difference in the spin relaxation rate between **1** and **2**, we perform the first principles calculation on the spin polarization and corresponding electron orbitals using the generalized gradient approximation (GGA) of the Perdew, Burke, and Ernzerholf (PBE) form[12] under package CASTEP[13] in which a plane-wave norm-conserving pseudopotential Method[14] is adopted. The obtained spins contributed from Nd *f* states as functions of energy for structures **1** and **2** are plotted in Figure S17. From the curves we can see that the spin states in structure **1** are connected to continuous bands, while the spin states in structure **2** are more isolated to separated levels. This implies that the states in **1** are more extended in the space and the states in **2** are more isolated to the Nd atoms. The extended states are more easily influenced by the environment, especially by the spin relaxation processes due to the spin-orbit interaction plus phonons. For the sake of illustration in Figure 5 we plot the spatial distributions of the spin states at the Fermi level in Nd atom and its 4 ligands for both **1** and **2**. Compared with the extended feature of the states in **1**, the states in **2** are so isolated that they even could not be extended to the nearest ligands of the Nd atom.

Supplemental References

1. Wada, H.; Ooka, S.; Yamamura, T.; Kajiwara, T. Light Lanthanide Complexes with Crown Ether and Its Aza Derivative Which Show Slow Magnetic Relaxation Behaviors. *Inorg. Chem.* **2017**, 56, 147-155.
2. Takehara, C.; Then, P. L.; Kataoka, Y.; Nakano, M.; Yamamura, T.; Kajiwara, T. Slow Magnetic Relaxation of Light Lanthanide-based Linear $LnZn_2$ Trinuclear Complexes. *Dalton Trans.* **2015**, *44*, 18276-18283.
3. Singh, S. K.; Gupta, T.; Ungur, L.; Rajaraman, G. Magnetic Relaxation in Single-Electron Single-Ion Cerium(III) Magnets: Insights from Ab Initio Calculations. *Chem.-Eur. J.* **2015**, *21*, 13812-13819.
4. Le Roy, J. J.; Korobkov, I.; Kim, J. E.; Schelter, E. J.; Murugesu, M. Structural and Magnetic Conformation of a Cerocene $[Ce(COT'')_2]^-$ Exhibiting a Uniconfigurational f^1 Ground State and Slow-magnetic Relaxation. *Dalton Trans.* **2014**, *43*, 2737-2740.
5. Hino, S.; Maeda, M.; Yamashita, K.; Kataoka, Y.; Nakano, M.; Yamamura, T.; Nojiri, H.; Kofu, M.; Yamamuro, O.; Kajiwara, T. Linear Trinuclear Zn(ii)–Ce(iii)–Zn(ii) Complex Which Behaves as a Single-molecule Magnet. *Dalton Transactions* **2013**, *42*, 2683-2686.
6. Hino, S.; Maeda, M.; Kataoka, Y.; Nakano, M.; Yamamura, T.; Kajiwara, T. SMM Behavior Observed in $Ce(III)Zn(II)_2$ Linear Trinuclear Complex. *Chem. Lett.* **2013**, *42*, 1276-1278.
7. Khélifa, A. B.; Belkhiria, M. S.; Huang, G.; Freslon, S.; Guillou, O.; Bernot, K. Single-molecule Magnet Behaviour in Polynuclear Assembly of Trivalent Cerium Ions with Polyoxomolybdates. *Dalton Trans.* **2015**, *44*, 16458-16464.
8. Gupta, S. K.; Rajeshkumar, T.; Rajaraman, G.; Murugavel, R. An Unprecedented Zero Field Neodymium(iii) Single-ion Magnet Based on a Phosphonic Diamide. *Chem. Commun.* **2016**, *52*, 7168-7171.
9. Baldoví, J. J.; Clemente-Juan, J. M.; Coronado, E.; Duan, Y.; Gaita-Ariño, A.; Giménez-Saiz, C. Construction of a General Library for the Rational Design of Nanomagnets and Spin Qubits Based on Mononuclear *f*-Block Complexes. The Polyoxometalate Case. *Inorg. Chem*. **2014**, *53*, 9976-9980.
10. Le Roy, J. J.; Gorelsky, S. I.; Korobkov, I.; Murugesu, M. Slow Magnetic Relaxation in Uranium(III) and Neodymium(III) Cyclooctatetraenyl Complexes. *Organometallics* **2015**, *34*, 1415-1418.
11. (a) Madison, W. SAINT v5.0–6.01, Bruker Analytical X-ray Systems Inc, 1998. (b) Sheldrick, G. M. SADABSs: An Empirical Absorption Correction Program, 1996. (c) Patterson, A. L. A Fourier Series Method for the Determination of the Components of Interatomic Distances in Crystals. Phys. Rev. 1934, 46, 372-376. (d) SHELXTL 6.10, Bruker Analytical Instrumentation: Madison, WI, 2000.
12. Perdew, J. P.; Burke, K.; Ernzerhof, M. Generalized Gradient Approximation Made Simple. *Phys. Rev. Lett.* **1996**, *77*, 3865-3868.
13. Clark, S. J.; Segall, M. D.; Pickard, C. J.; Hasnip, P. J.; Probert, M. J.; Refson, K.; Payne, M. C. First Principles Methods Using CASTEP. *Z. Kristallogr.* **2005**, *220*, 567-570.
14. Hamann, D. R.; Schluter, M.; Chiang, C. Norm-Conserving Pseudopotentials. *Phys. Rev. Lett.* **1979**, *43*, 1494-1497.
